# Experimental and Theoretical Biological Probing of Schiff Bases as Esterase Inhibitors: Structural, Spectral and Molecular Insights

**DOI:** 10.3390/molecules28155703

**Published:** 2023-07-28

**Authors:** Muhammad Asam Raza, Muhammad Waseem Mumtaz, Seyhan Öztürk, Muhammad Latif, Aisha, Adnan Ashraf, Necmi Dege, Onur Erman Dogan, Erbil Agar, Shafiq Ur Rehman, Awal Noor

**Affiliations:** 1 Department of Chemistry, Hafiz Hayat Campus, University of Gujrat, Gujrat 50700, Pakistan; muhammad.waseem@uog.edu.pk (M.W.M.); aishafatima408@gmail.com (A.); 2 Department of Chemistry, Faculty of Sciences, Ondokuz Mayıs University, Samsun 55139, Türkiye; sturna@omu.edu.tr (S.Ö.); oedogan@omu.edu.tr (O.E.D.); erbagar@omu.edu.tr (E.A.); 3 Department of Biochemistry and Molecular Medicine, College of Medicine, Taibah University, Madinah 42318, Saudi Arabia; 4 Centre for Genetics and Inherited Diseases (CGID), Taibah University, Madinah 42318, Saudi Arabia; 5 Department of Chemistry, University of Lahore, Lahore 54000, Pakistan; adnan.ashraf@chem.uol.edu.pk; 6 Department of Physics, Faculty of Sciences, Ondokuz Mayıs University, Samsun 55139, Türkiye; necmi.dege@omu.edu.tr; 7 Department of Chemistry, University of Central Punjab, Lahore 54590, Pakistan; shafiqchemist@gmail.com; 8 Department of Basic Sciences, Preparatory Year Deanship, King Faisal University, Al Hassa 31982, Saudi Arabia

**Keywords:** Schiff base, molecular docking, crystal structure, density functional theory, Hirshfeld surface analysis

## Abstract

The present study was designed to evaluate the in vitro and in silico potential of the Schiff bases (*Z*)-4-ethoxy-*N*-((5-nitrothiophen-2-yl)methylene)benzenamine (**1**) and (*Z*)-2,4-diiodo-6-((2-methyl-3-nitrophenylimino)methyl)phenol (**2**). These Schiff bases were synthesized according to a reported method using ethanol as a solvent, and each reaction was monitored on a TLC until completion of the reaction. The structures of both compounds were elucidated using spectroscopic techniques such as UV–Vis, FTIR, ^1^H NMR and ^13^C NMR. Molecular structure was determined using single-crystal XRD, which revealed that compounds **1** and **2** were monoclinic and triclinic, respectively. Hirshfeld surface analysis (HS) and 2D fingerprint plots were used to determine the intermolecular interactions along the contact contribution in the crystalline molecules. The structures of both compounds were optimized through a hybrid functional method B3LYP using the 6-31G(d,p) basis set, and various structural parameters were studied. The experimental and theoretical parameters (bond angle and bond length) of the compounds were compared with each other and are in close agreement. The in vitro esterase potential of the synthesized compounds was checked using a spectrophotometric model, while in silico molecular docking studies were performed with AutoDock against two enzymes of the esterase family. The docking studies and the in vitro assessment predicted that such molecules could be used as enzyme inhibitors against the tested enzymes: acetylcholine esterase (AChE) and butyrylcholine esterase (BChE).

## 1. Introduction

Schiff bases (SB) are synthesized by the reaction of aldehydes/ketones and amines in a suitable medium and have shown many valuable pharmacological applications, including the inhibition of acetylcholine esterase and butyrylcholine esterase, enzymes responsible for Alzheimer’s disease [1,2,3]. SBs also behave as ligands that develop coordination with metals through imine nitrogen [4]. Characteristically, geometrical cavity restrictions can be provided by SBs, which are important for host–guest cooperation and lipophilicity modulation [5]. These exceptional properties offer stability, selectivity, and sensitivity to the SB. Chemists are still trying to formulate various types of SB installed with diverse structural features for key applications, and well-designed and effective ligands of SBs are contemplated as “privileged ligands” [6]. SBs are extensively engaged in catalysis, separation phenomena, biochemistry, material science, decarboxylation, and enzymatic aldolization [7,8]. Heterocyclic cores are one of the dominant organic classes containing at least one hetero atom in the ring [8]. The general structures of heterocyclic and cyclic organic compounds exhibit similarities, while the existence of the heteroatoms administers some sort of chemical and physical properties in these heterocyclic compounds [8,9]. Many of them are those heterocycles that contain six- and five-membered rings in their nucleus, and these compounds mostly have one to three atoms that are hetero in nature [10]. It is recognized that due to the compressed structure of many heterocyclic compounds, they acquire different properties, such as anti-wear, anti-corrosion, and anti-oxidant [11] traits. In the cells of a living organism, heterocycles display a fundamental role [10]. These compounds have vast dimensional applications in different fields, as they are potent among those categorical compounds that have been used as veterinary products, pharmaceuticals, and agrochemicals. Heterocyclic compounds also exhibit applications such as dyestuffs, sanitizers, antioxidants, developers, copolymers, and corrosion inhibitors [12].

In drug discovery, the molecular docking technique has emerged as an increasingly significant tool. A docking study can be utilized to represent an interaction between a protein and a small ligand molecule at its atomic level [13]. This venture allows researchers to identify the behavior of small molecules regarding connectivity at the binding site of a target protein and illustrates their fundamental biochemical pathways. This tool further characterizes the ‘‘best-fit’’ ligand orientation that binds with a specific protein molecule and can be used to conclude the intermolecular structure of the complex organized among more than two molecules [14]. Due to its large number of medicinal applications, the case of ligand–protein interaction has become much more fascinating. A ligand behaves like a small molecule that exhibits interaction at the specific site of a protein. Various binding modes have considerable mutual conformations, through which binding phenomena could occur. Before the process of the docking study, knowledge about the binding site may significantly enhance its efficiency [15]. In this work, we have synthesized two novel SBs in continuation of our previous work. The structures of the compounds were determined with SCXRD; computation studies were done with Gaussian software; while biological potentials were carried out using in vitro and in silico approaches.

## 2. Results and Discussion

### 2.1. Spectroscopic Studies of the Synthesized Compounds (***1***–***2***)

This current study is an extension of an already reported study of compounds by our research group [1,3,16]. In this project, we have synthesized two new SBs through the condensation of 5-nitro-2-thiphenecarboxaldehyde with *p*-phenetidine and 2-(trifluoromethyl)aniline under reflux conditions [1,16]. The progress of the reaction was monitored on a TLC, and after completion of the reaction, the compounds were crystallized in ethanol under slow evaporation at room temperature. The structure of the targeted compounds was elucidated using spectroscopic techniques and X-ray diffraction. Compound **1** showed two absorption bands at 286 nm and 416 nm, while compound **2** displayed them at 230 nm, 235 nm, 286 nm, and 372 nm under UV–Vis spectroscopy (Figures S1 and S2). In UV–Vis, the band below 300 nm was attributed to the *π*–π* transition, and the band above 300 nm was assigned to *n*-π* transitions [17]. On the FTIR spectrum, various informative bands appeared in the range of 650–4000 cm^−1^. The absorption band in the range of 1690–1590 cm^−1^ is a characteristic band of the C=N group in a Schiff base and considered as a confirmatory indicator for the formation of an SB [18,19]. In FTIR spectra of compounds **1** and **2**, strong bands appeared at 1609 cm^−1^ and 1518 cm^−1^, respectively, and were ascribed to the presence of azomethine linkage. The appearance of such bands in **1** and **2** gave a preliminary indication regarding the targeted products. Furthermore, due to the presence of an iodine (I) group in compound **2**, the C=N group was shifted to 1495 cm^−1^ as compared to **1**. The IR bands at 1531 cm^−1^ and 1595 cm^−1^ were due to C=C (aryl) in **1** and **2,** respectively (Figures S3 and S4).

NMR spectra were recorded in DMSO-*d_6_* using a Bruker Avance III 400 MHz NMR spectrometer. The disappearance of NH_2_ and the appearance of a new singlet peak in ^1^H-NMR were assigned to HC=N at δ 8.90 and 13.81, which confirmed the synthesis of the targeted compounds, respectively [20]. The doublet peaks at δ 8.17 and 7.65 with coupling constant 4.3 Hz were due to the protons of the thiophene moiety in compound **1**. The signals for Ar-H in **1** and **2** were observed in the range δ 7.46–6.84 and 7.27–8.38, respectively (Figures S5 and S6). The signals of methylene and the methyl group in **1** were exhibited at 4.06 and 1.33 ppm, respectively. The ^13^C NMR of **1** and **2** showed eleven and fourteen signals from 158.14 to 14.63 ppm and 162.66 ppm to 14.25 ppm, respectively. All these peaks suggested the formation of **1** and **2,** as shown in Figures S7 and S8.

Yield: 83%; m.p.: 142 °C; λ_max_: 286 nm and 416 nm; IR: 3087 (C–H, aromatic), 2984 (C–H, aromatic), 1609 (HC=N), 1574 (C–NO_2_), 1531 (C=C), 1118 (C–O), 839 (C–S) cm^−1^; ^1^H NMR (400 MHz, DMSO-*d*_6_): δ 8.90 (s, 1H), 8.17 (d, *J* = 4.3 Hz, 1H), 7.65 (d, *J* = 4.3 Hz, 1H), 7.46–7.25 (m, 1H), 7.07–6.84 (m, 3H), 4.06 (q, *J* = 6.9 Hz, 2H), 1.33 (t, *J* = 7.0 Hz, 3H); ^13^C NMR (100 MHz, DMSO-*d*_6_): δ 158.46, 151.94, 150.43, 149.40, 141.82, 131.04, 130.53, 123.35, 115.06, 63.38, 14.63.

Yield: 90%; m.p.: 162 °C; λ_max_: 230 nm, 235 nm, 286 nm, and 372 nm; IR: 3080 (C–H, aromatic), 1518 (HC=N), 1612 (C–NO_2_), 1595 (C=C), 1148 (C–O); ^1^H NMR (400 MHz, CDCl_3_): δ 13.82 (s, 1H), 8.40 (s, 1H), 8.17 (d, *J* = 2.0 Hz, 1H), 7.79 (d, *J* = 8.1 Hz, 1H), 7.74 (d, *J* = 2.1 Hz, 1H), 7.44 (t, *J* = 8.0 Hz, 1H), 7.29 (d, *J* = 8.0 Hz, 1H), 2.54 (s, 3H).; ^13^C NMR (100 MHz, CDCl_3_): δ 162.66, 159.99, 151.34, 150.13, 148.84, 141.07, 127.56, 127.15, 122.91, 122.59, 120.56, 87.27, 80.65, 14.25.

### 2.2. XRD Analysis of the Synthesized Compounds (***1***–***2***)

**Figure 1:**
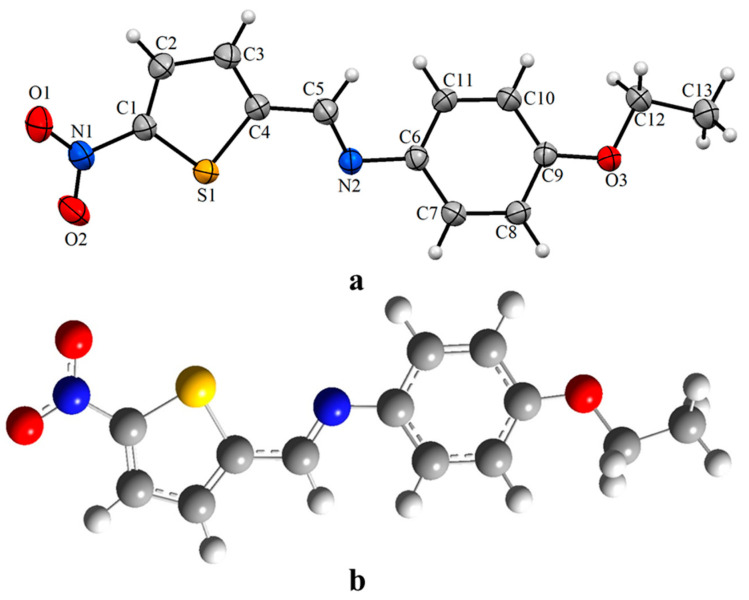
(**a**) Crystal unit; (**b**) optimized structure of compound **1**.

**Figure 2:**
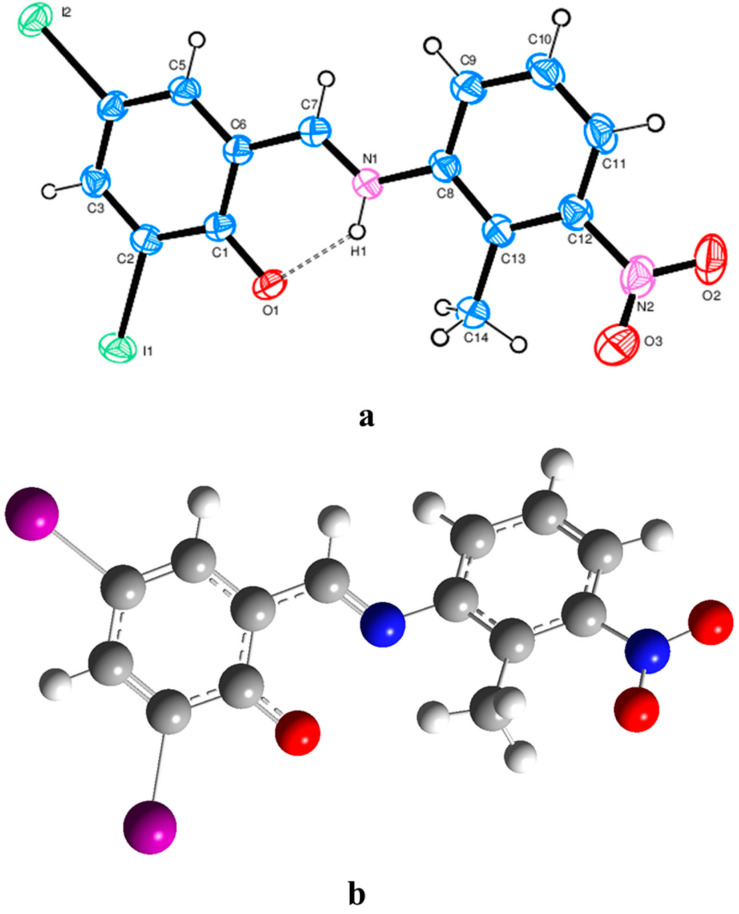
(**a**) Crystal unit; (**b**) optimized structure of compound **2**.

The structure of compounds **1** and **2** was further confirmed by the single-crystal X-ray analyses (Tables S1–S8). Table 1 summarizes the experimental details (crystal data, data collection, and structure refinement). Figure 1 and Figure 2 show the asymmetric unit of **1** and **2**, respectively. Compound **1** has one independent molecule in the asymmetric unit. In 1, the C6–C11 benzene ring is inclined to the thiophene ring by 33.86(8)°, while the corresponding angle for C7–C12 is 37.22(12)°. The molecular structure is stabilized by the intermolecular C5—H5∙∙∙O2 (x, y, −z + 1) hydrogen bond along the b-axis direction in **1** (Figures S9 and S10 and Table S9). In compound **1**, the chains are linked by C12—H12B∙∙∙Cg2 (−x + 1, −y + 1, −z + 1) and very weak π-stacking interactions [Cg2∙∙∙Cg2 = 3.6134(2) Å. Cg2 is the center of the C6–C11 ring], forming a three-dimensional network. In the methoxy groups in **1**, the C9—O3 and C12—O3 bond distances are a little different, 1.370(3) Å and 1.438 (3) Å, respectively. The N–bound H atom was located in a difference Fourier map and refined with Uiso(H) = 1.2 UeqN) and a distance restraint. The C-bound H atoms were positioned geometrically (C—H = 0.93, 0.96, and 0.97 Å) and refined using a riding model, with Uiso(H) = 1.5 Ueq(C) for methyl H atoms and 1.2 Ueq(C) for other H atoms [21,22,23,24]. The detailed geometric parameters of **1** and **2** are given in Tables S10–S13.

### 2.3. Computational Studies

#### 2.3.1. Frontier Molecular Orbitals

**Figure 3:**
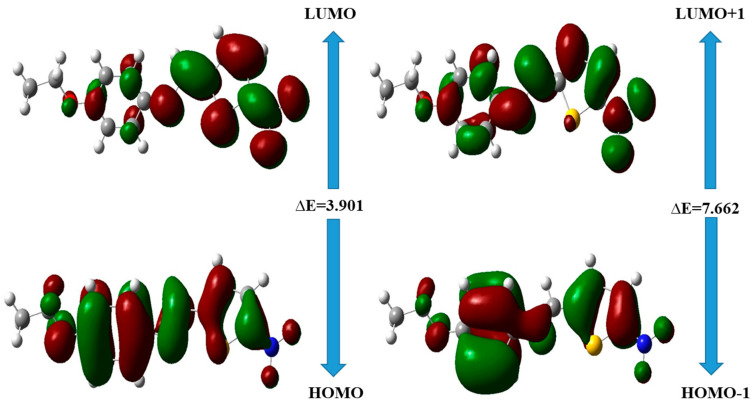
HOMO and LUMO of Compound **1**.

**Figure 4:**
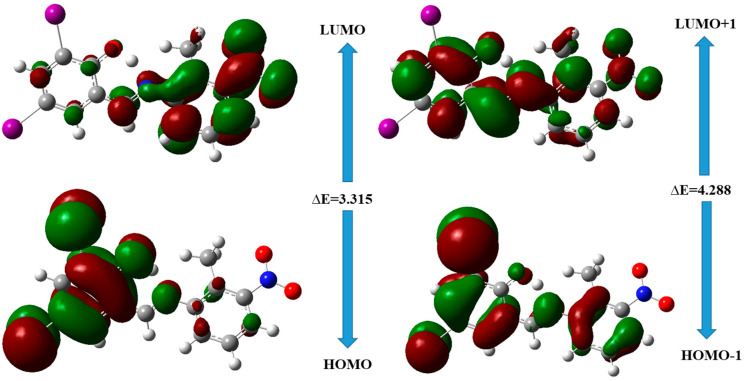
HOMO and LUMO of compound **2**.

The optimized structures of the compounds under study were presented in Figure 1 and Figure 2. The bond length and bond angle of the synthesized compounds were compared between experimental (XRD) data and theoretical (DFT) results. It was determined from comparison that both studies (experimental and theoretical) have close agreement with each other, as shown in Tables S9–S13. Furthermore, the relationship between the DFT and XRD calculations was further evaluated on the basis of a correlation coefficient (R^2^). It was seen that the R^2^ value is 0.9886 and 0.9679 for bond length and bond angle, respectively, for compound **1**, while for **2**, it is 0.9922 and 0.8098 for bond length and angle, respectively, as shown in Figures S11–S14. These results demonstrated that in both studies the structural parameters are very close to each other as the value of the correlation coefficient is close to 1.0. The slight difference of the bond angle in compound **1** may be due to the gaseous and solid phase calculation of the DFT and XRD studies, respectively. The DFT and XRD results were further compared by superimposing the structures of each compound (Figure S14a,b). The root mean square deviation (RMSD) was calculated after superimposing the geometries of the compounds. The RMSD was 0.2427 and 0.8398 for compound **1** and **2**, respectively. A DFT study was also used to draw the HOMO and LUMO orbital of both compounds along energy gaps between different orbitals. The energy difference between the HOMO and LUMO of compound **1** is 3.901 eV, while HOMO−1 and LUMO+1 is 7.662 eV, as shown in Figure 3. The HOMO and LUMO energy difference of **2** is 3.315 eV, and similarly, HOMO−1 and LUMO+1 is 4.288 eV (Figure 4). The energy difference between the HOMO and LUMO of both compounds is relatively enough to stabilize the molecules [25]. The chemical reactivity of the molecules depends on the HOMO–LUMO energy gap, and molecules may be differentiated as hard or soft on the basis of the HOMO–LUMO energy gap. A molecule having a small energy gap is denoted as a soft molecule, and a molecule with a large energy gap is denoted as hard. The molecule with a small energy gap has a strong ability to demonstrate good biological activity because it requires little energy for excitation. Furthermore, compounds in which the LUMO is more stabilized also display excellent activity in a biological system. In our study, as compound **2** has a small energy gap that is also due to the presence of iodine (electronegative) atoms on the ring, it displayed higher activity as compared to compound **1**.

#### 2.3.2. Natural Bond Orbitals

The natural bond orbitals (NBO) of the synthesized compounds were computed with Gaussian software. NBOs are used in finding the individual bond in the molecules and their energies linked with bond pair/lone pair electrons, which helps in the interactions of atoms. With the use of NBOs, we can predict the behavior of the donor and acceptor atom in a molecule. During such interaction, electrons lose occupancy from a bonded orbital and shift to an empty orbital. It was found in compound **1** under study that S1-C17 behaved as a donor and that C28 acted as an acceptor with the energy of 0.51 kcal/mol, while O2-C20 and C24 had the energy of 0.59 kcal/mol. The energy associated between N3 to C7 is 2.60 kcal/mol, and that of O4-N5 to C23 is 0.63 kcal/mol. The NBOs of compound **2** are also listed in Table S14, along with the highest energy assisted with I1-C19 to C12, which is 1.77 kcal/mol, while the least energy (0.52 kcal/mol) is N4-C18 to O6. Similarly, the energy is 11.87 kcal/mol when N4-O5 behaves as a donor and O6 behaves as an acceptor.

#### 2.3.3. Density of State

The density of state (DOS) of the synthesized compounds was computed from the optimized files of the targeted compounds using GaussSum software. It is an important strategy in order to determine the different state or energy levels in the molecules for the excitation of the electrons from the ground state. It was clear from DOS spectra as shown in Figure S15 that energy differences between HOMO−1 and LUMO+1 varied among both compounds. The energy difference between HOMO to LUMO and HOMO−1 to LUMO+1 for compound **1** is greater than that of compound **2**. It is also calculated from the FMOs of compound **1** that the energy gap for HOMO/LUMO is 3.901 eV, while HOMO−1/LUMO+1 is 7.662 eV, and the overall energy difference is 3.761 eV. The energy difference between HOMO/LUMO and HOMO−1/LUMO+1 is 3.315 eV and 4.288 eV, respectively, for compound **2**. The net difference in the form of energy among FMOs is 0.973 eV.

#### 2.3.4. Global Reactivity Parameters

The global reactivity parameters of the synthesized compounds were calculated from the output files of the synthesized compounds with Gaussian software. These parameters are important in order to understand the reactivity as well as stability of the compounds. Compound **1** has high electronegativity as compared to compound **2**, which is due to the presence of -OH in the molecule. The ionization and electron affinity of compound **1** are 8.294 and 6.1168, respectively (Table S15). The chemical hardness and chemical potential are favorable for the kinetic stability of the compounds under study.

### 2.4. Hirshfeld Surface Analysis

**Figure 5:**
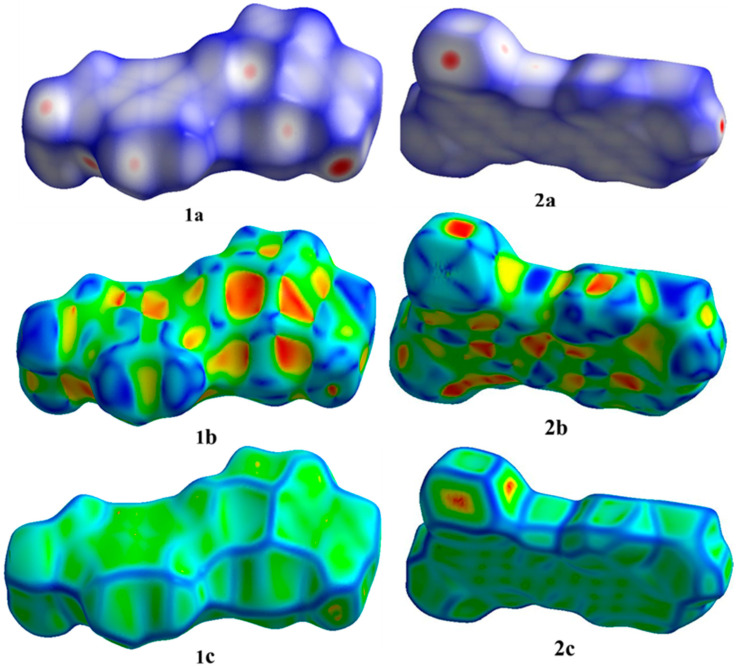
Hirshfeld surfaces mapped at three views: (**a**) *d*norm, (**b**) shape index, and (**c**) curvature.

Hirshfeld surface analysis (HS) is mostly used to check the intermolecular interactions in the crystalline compounds, which stabilize the crystals. The *di* (inside) and the *de* (outside) demonstrate the distances to the Hirshfeld surface beyond the nuclei in the context of van der Waals radii. The HS is mapped with white, red, and blue colors contingent upon the distances of the total radii [16,26]. The Hirshfeld surfaces of the compounds were created using a high surface resolution with the 3D *d_norm_* surfaces and mapped over a *d_norm_* range of −0.55 to 1.0 Å, a shape-index range of −0.10 to 1.0 Å, and curvedness ranged from −0.40 to 4.0 Å. The surfaces were made to seem non-transparent to show a picture of the molecules, around which they were calculated. For the identification of close contacts, the *d_norm_* surface was used, and its value ranged from negative to positive. The negative value represented the shorter contacts, while positive value depicted longer contacts compared to *r^vdW^* (van der Waals radii). The red area meant closer contacts with a negative *d_norm_* value; blue spots on surfaces exhibited longer contacts and a positive value *of d_norm_*; while white colors on the surfaces were due to equal distance along a zero value (Figure 5).

Moreover, 2 D fingerprint plots of the compounds under study were mapped to check the connections between atoms, as shown in Figures S16 and S17. It was observed from contacts that H….H (28.8%) interactions are major contributors in compound **1,** while in compound **2**, it is H….F/F….H (26.7%). O….H/H….O are the second major contributing contacts in both compounds. The remaining contacts in compound **1**, which stabilize the compound, are H…C/C…H, H…S/S…H, H…N/N…H, and C…O/O…H, with percentage 15.9, 10.0, 7.5, and 5.9, respectively, as shown in Figure S16. Compound **2** has displayed the contacts O…H/H…O (20.8%), I…H/H…I (20.7%), H…H/H…H (17.5%), C…H/H…C (9.2%), I…O/O…I (7.9%), and I…C/C…I (3.3%).

### 2.5. In Vitro and In Silico Enzyme Inhibition

**Figure 6:**
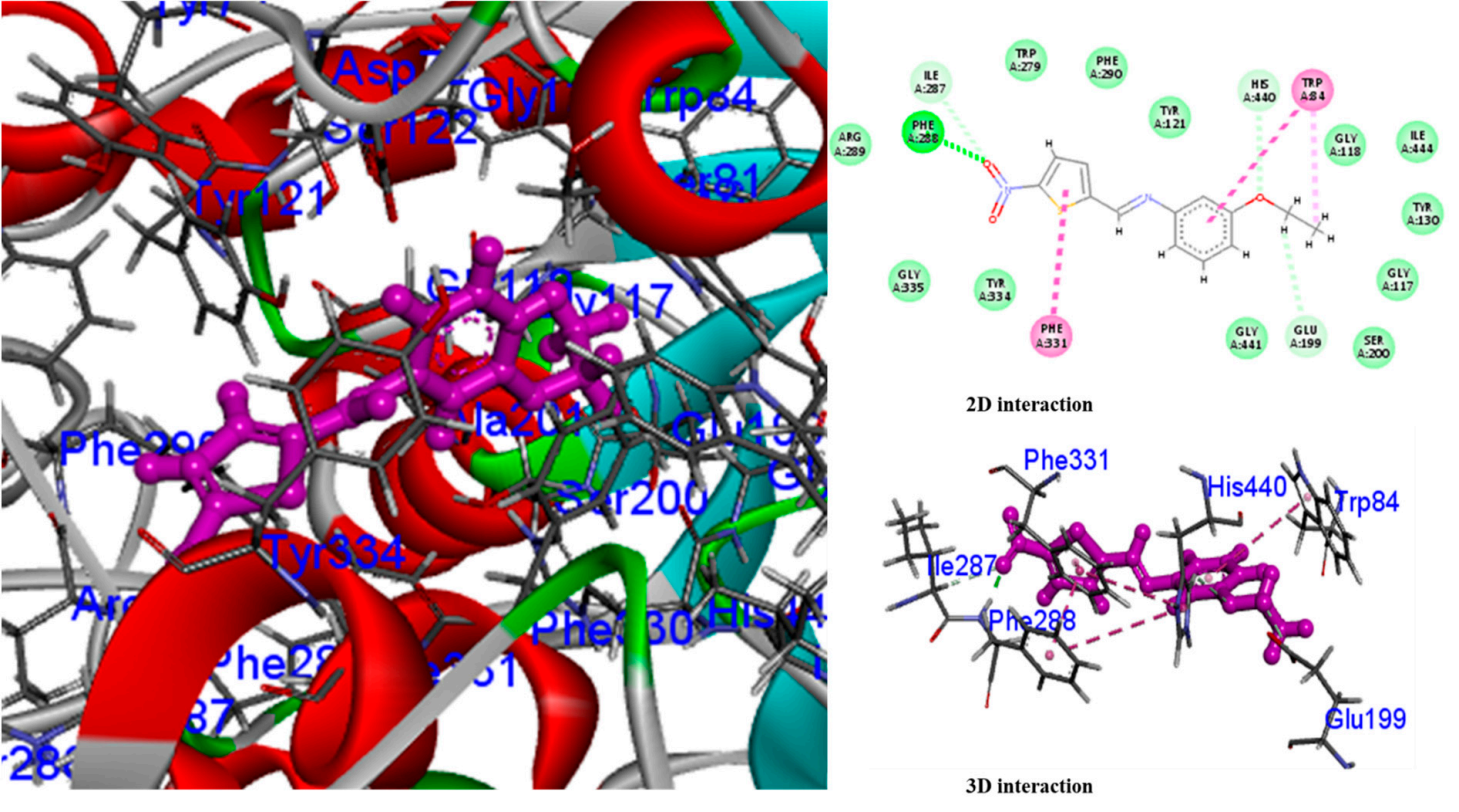
Docking pose of compound **1** with AChE.

The esterase family basically belongs to the hydrolase enzyme and actively participates in Alzheimer’s disease. The in vitro potential of the synthesized compounds is presented in Table 2. It was found that compound **2** was more active in AChE as well as against BChE. Docking approaches frequently account for an energy-based scoring function, which helps to analyze the most affirmative conformation of ligands when binding with the target [15]. From general hypothesis, it is illustrated that the lesser the energy score value, the better the binding of a protein–ligand molecule, compared to those with greater energy values [27]. Therefore, it is necessary to identify those ligand-binding modes that have the lowest energy value [28]. The docking studies of the synthesized compounds were carried out using standard parameters. From docking study, it was determined that SBs can be used as enzyme inhibitors, as they exhibited remarkable docking scores as well as binding energy (Table 2). It was concluded from the results presented in the table that compound **2** exhibited the highest dock score as compared to compound **1** against AChE, while against BChE, a similar pattern was shown by both compounds. Compound **1** showed interactions with AChE with π-π and π-alkyl interaction with Trp84. Moreover, π-π interactions were also exhibited by compound **1** with Phe331, while with Phe288 it showed strong hydrogen bonding interaction due to the oxygen atom of the nitro group. Furthermore, compound **1** also exhibited interactions with Ile287, His440, and Glu199, as shown in Figure 6. On the active site of BChE, compound **1** exhibited interaction with Trp82 via π-π interaction with phenyl as well as the thiophene ring of compound **1**. Compound **1** also showed interactions with BChE and may have inhibited BChE due to hydrogen bonding interactions (Figure S18). Compound **2** also showed a good docking score as well as binding energy with AChE (docking score: −12.8735) and BChE (docking score: −11.1704). The phenyl ring of compound **2** exhibited π-π interaction with Trp84 and Trp334, while the nitro group on aniline showed hydrogen bond interaction with Gly117, His440, Gly118, Glu199, and Tyr130. Similarly, the hydroxyl group on the phenyl ring showed hydrogen bond interaction with Asp72, as shown in Figure S19. The iodine atoms on molecule **2** showed interactions with Phe330 and Tyr70 on AchE (Figure S19). Compound **2** demonstrated firm binding with different amino acid residues located on the active site of BChE with Trp82, Glu197, Gly116, Gly115, and Tyr332. The compound showed hydrogen bond interaction with Glu197, Tyr128, Gly115, and Gly116. In contrast to hydrogen bonding interactions, compound **2** also showed interaction with Tyr332, viz., strong π-alkyl and π-π interaction between the phenyl rings of Schiff base **2** and the phenyl ring of Trp82 (Figure S20).

## 3. Materials and Methods

### 3.1. Chemicals and Instruments

The 5-Nitro-2-thiphenecarboxaldehyde, *p*-phenetidine, 2-(trifluoromethyl)aniline, ethanol, and all other solvents and reagents used in this study were purchased from Merck (Germany). UV–Vis absorption spectra were obtained at room temperature in a region of 200 to 900 nm for a 1 × 10^−3^ M solution of the sample in ethanol, using a Thermo Evolution Array UV–Vis spectrophotometer. A PerkinElmer Spectrum Two FT-IR spectrophotometer equipped with an ATR module was used to carry out the IR spectra of the prepared compounds in a region from 4000–400 cm^−1^. NMR spectra were recorded in DMSO-d_6_ using a Bruker Avance III 400 MHz NMR Spectrometer.

### 3.2. Synthesis of (Z)-4-Ethoxy-N-((5-nitrothiophen-2-yl)methylene)benzenamine (***1***) and (Z)-2,4-Diiodo-6-((2-methyl-3-nitrophenylimino)methyl)phenol (***2***)

**Scheme 1:**
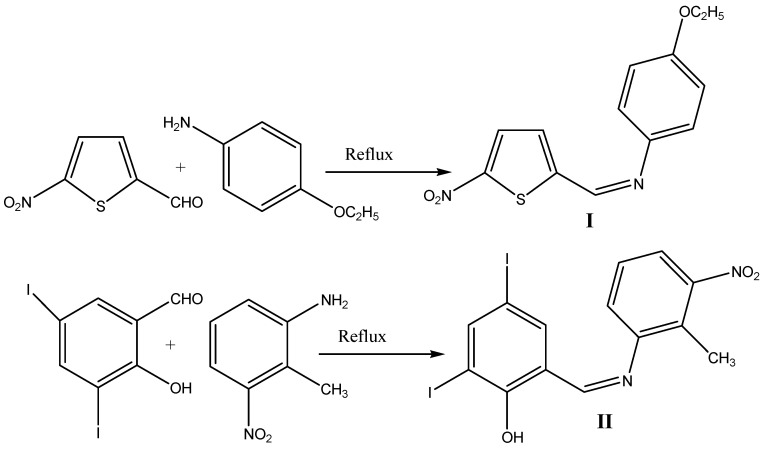
Synthetic scheme of SB.

The title compounds (**1** and **2**) were prepared according to the protocol reported by our research group [1,16]. The SBs were synthesized through the condensation of an equimolar mixture of 5-nitro-2-thiphenecarboxaldehyde with *p*-phenetidine that yielded **1**, while condensation of 2-hydroxy-3,5-diiodobenzaldehyde with 2-methyl-3-nitrobenzenamine yielded **2** in acetonitrile under reflux for 36 h (Scheme 1). Reaction progress was monitored on a TLC at regular intervals. After completion of the reaction, a burgundy product was obtained, which was filtered off, and crystals of the respective compounds were obtained by slow evaporation using ethanol at room temperature.

### 3.3. Crystal Structure Determination

X-ray single-crystal diffraction data were collected at 296 K for **1** and **2** on a STOE *IPDS* 2 [29] diffractometer equipped with a graphite monochromator using Mo-Kα radiation (λ = 0.71073 Å). The structures were solved by direct methods using the *SHELXT* program [30] and refined on *F*^2^ by full matrix least squares using the *SHELXL* program [31]. Unit cell refinement using all observed reflections and data reduction were performed using *X-AREA*. All non-hydrogen atoms were refined anisotropically, and the hydrogen atoms were included in geometric positions. The final difference Fourier maps showed no peaks of chemical significance. The molecular geometry calculations and drawings were performed with WinGX [32].

### 3.4. Computational Studies

Optimization and DFT study were conducted with Gaussian 09 using the hybrid functional B3LYP method 6-31G(d,p) basis set [33,34]. The results of DFT studies in terms of frontier molecular orbitals (FMO), natural bond orbitals (NBO), density of state (DOS), and global reactivity parameters were viewed in GaussView 5.0. For optimization purposes, the input files were taken from the crystal structure of the respective molecule to achieve the best match with the experimental data [1,16,35].

### 3.5. Hirshfeld Surfaces Analysis

A Hirshfeld surface is an outer contour of the space a molecule or an atom consumes in a crystalline environment. The Hirshfeld surfaces along with 2D fingerprint plots were calculated using the Crystal Explorer 17.5 program with built in TONTO [35]. The normalized contact distance *d_norm_* based on both *de* and *di* were calculated using a standard equation [1,36].

### 3.6. In Vitro and In Silico Assessments Involving Esterases

In vitro assessment was carried out using spectrophotometric method [37]. First, 100 µL of the tested compound (5 mg/mL) was added in a test tube, followed by the addition of 100 µL enzymes (AChE and BChE separately); the contents were mixed and incubated for 10 min at 37 °C. After 10 min of incubation, a 0.5 mL buffer (50 mM), 50 µL of AChI, and DTNB were added. The test tubes were incubated at 37 °C for 30 min, and after incubation, absorbance was measured at 410 nm using a UV–Vis spectrophotometer. The PMSF was used as a positive control (standard), while DMSO was used as a negative control during activity. The IC_50_ values were calculated using various concentrations (100–10 µL) of the tested compounds. The docking studies of the title compounds were carried out with AutoDock, an online freely available software docking program [1,37]. The crystal structures of AChE (acetylocholino esterase) and BChE (butyrylcholino esterase), with PDB codes 1EVE and 1P0I, respectively, were selected for docking. The docking results, interactions, and the analyses of surfaces in graphical form were viewed using Discovery Studio Visualizer.

## 4. Conclusions

Organic chemists are continuously synthesizing new bioactive molecules for the betterment of human health. SBs have immense potential and active ingredients for curing many ailments related to human beings. In this work, we synthesized two novel Schiff bases comprising heterocyclic moieties, and structural elucidation was carried out using spectroscopic techniques. The assigned crystalline structure of each compound was further studied under Hirshfeld surface analysis. The 2D and 3D plots of compound **1** and **2** revealed crystalline stability due to various interactions. The crystal structures were further optimized with Gaussian software, and the calculations were compared with XRD results, which demonstrated that bond angle and bond length are in close agreement. With the use of Gaussian software, various structural parameters of the synthesized compounds were computed, such as electronegativity, electron affinity, chemical potential, and chemical hardness, which exhibited that these molecules have remarkable kinetic stability. The compounds were evaluated using in vitro and in silico models against AChE and BChE. Compound **2** demonstrated good results compared to **1**, as it showed 76.15 ± 1.2% inhibition against AChE and 72.32 ± 0.9% against BChE. The theoretical and experimental results revealed that both compounds had good potential to inhibit the enzymes, and they may be used as enzyme inhibitors to treat Alzheimer’s disease.

## Figures and Tables

**Table 1:** Crystal data and structure refinement of synthesized compounds.

Crystal Data	1	2
CCDC	2,075,472	2,258,087
Empirical formula	C_13_H_12_N_2_O_3_S	C_14_H_10_I_2_N_2_O_3_
Formula weight	276.318	508.04
Temperature/K	296	293 (2)
Crystal system	monoclinic	monoclinic
Space group	P2_1_/c	P2_1_/n
a/Å	11.9183 (6)	9.8544 (9)
b/Å	13.8575 (9)	12.5885 (7)
c/Å	8.0694 (5)	12.7810 (13)
α/°	90	90
β/°	103.195 (4)	101.501 (7)
γ/°	90	90
Volume/Å^3^	1297.54 (14)	1553.7 (2)
Z	4	4
ρ_calc_g/cm^3^	1.414	2.172
μ/mm^−1^	0.255	4.058
F (000)	576.8	952.0
Crystal size/mm^3^	0.76 × 0.353 × 0.08	0.545 × 0.424 × 0.354
Radiation	Mo Kα (λ = 0.71073)	MoKα (λ = 0.71073)
2Θ range for data collection/°	4.58 to 52	4.588 to 64.03
Index ranges	−18 ≤ h ≤ 18, −21 ≤ k ≤ 21, −10 ≤ l ≤ 12	−14 ≤ h ≤ 14, −18 ≤ k ≤ 18, −19 ≤ l ≤ 11
Reflections collected	17,422	13,542
Independent reflections	17,422 [R_int_ = 0.1318, R_sigma_ = 0.1114]	5312 [R_int_ = 0.0359, R_sigma_ = 0.0306]
Data/restraints/parameters	17,422/0/174	5312/1/192
Goodness-of-fit on F^2^	0.706	1.044
Final R indexes [I >= 2σ (I)]	R_1_ = 0.0529, wR_2_ = 0.1608	R_1_ = 0.0462, wR_2_ = 0.0944
Final R indexes [all data]	R_1_ = 0.0816, wR_2_ = 0.1988	R_1_ = 0.0841, wR_2_ = 0.1141
Largest diff. peak/hole/e Å^−3^	0.35/−0.42	0.80/−0.96

**Table 2:** In vitro and in silico assessment of the synthesized compounds.

Compounds	Docking Score (Kcal/mol)			Enzyme Inhibition		
	AChE	BChE	AChE		BChE	
			Inhibition (%)	IC_50_ (µg/mL)	Inhibition (%)	IC_50_ (µg/mL)
**1**	−11.0053	−10.3133	72.27 ± 0.9	193.6 ± 2.4	69.11 ± 1.3	209.4 ± 2.7
**2**	−12.8735	−11.1704	76.15 ± 1.2	184.1 ± 1.9	72.32 ± 0.9	214.6 ± 2.1
**Std**	-	-	81.52 ± 0.8	117.6 ± 1.4	74.01 ± 1.0	143.2 ± 1.9

## Data Availability

Data is available on request from corresponding author.

## Supplementary Materials

The following supporting information can be downloaded at: https://www.mdpi.com/article/10.3390/molecules28155703/s1, Spectral, DFT and XRD data.
