# Supplementary material for: Experimental and Theoretical Biological Probing of Schiff Bases as Esterase Inhibitors: Structural, Spectral and Molecular Insights
Source: Molecules. 2023 Jul 28;28(15):5703. doi: 10.3390/molecules28155703 (PMC10419919; doi:10.3390/molecules28155703)

Supplementary Materials

Experimental and Theoretical Biological Probing of Schiff Bases as Esterase Inhibitors: Structural, Spectral and Molecular Insights

Muhammad Asam Raza [1,*], Muhammad Waseem Mumtaz [1], Seyhan Öztürk [2], Muhammad Latif [3,4], Aisha [1], Adnan Ashraf [5], Necmi Dege [6], Onur Erman Dogan [2], Erbil Agar [2], Shafiq Ur Rehman [7] and Awal Noor [8,*]

[1] Department of Chemistry, Hafiz Hayat Campus, University of Gujrat, Gujrat 50700, Pakistan; muhammad.waseem@uog.edu.pk (M.W.M.); aishafatima408@gmail.com (A.)
[2] Department of Chemistry, Faculty of Sciences, Ondokuz Mayıs University, Samsun 55139, Türkiye; sturna@omu.edu.tr (S.Ö.); oedogan@omu.edu.tr (O.E.D.); erbagar@omu.edu.tr (E.A.)
[3] Department of Biochemistry and Molecular Medicine, College of Medicine, Taibah University, Madinah 42318, Saudi Arabia
[4] Centre for Genetics and Inherited Diseases (CGID), Taibah University, Madinah 42318, Saudi Arabia
[5] Department of Chemistry, University of Lahore, Lahore 54000, Pakistan; adnan.ashraf@chem.uol.edu.pk
[6] Department of Physics, Faculty of Sciences, Ondokuz Mayıs University, Samsun 55139, Türkiye; necmi.dege@omu.edu.tr
[7] Department of Chemistry, University of Central Punjab, Lahore 54590, Pakistan; shafiqchemist@gmail.com
[8] Department of Basic Sciences, Preparatory Year Deanship, King Faisal University, Al Hassa 31982, Saudi Arabia
* Correspondence: asamgcu@yahoo.com (M.A.R.); anoor@kfu.edu.sa (A.N.)

Supplementary Data

Figure S1: UV/VIS spectrum of **1**

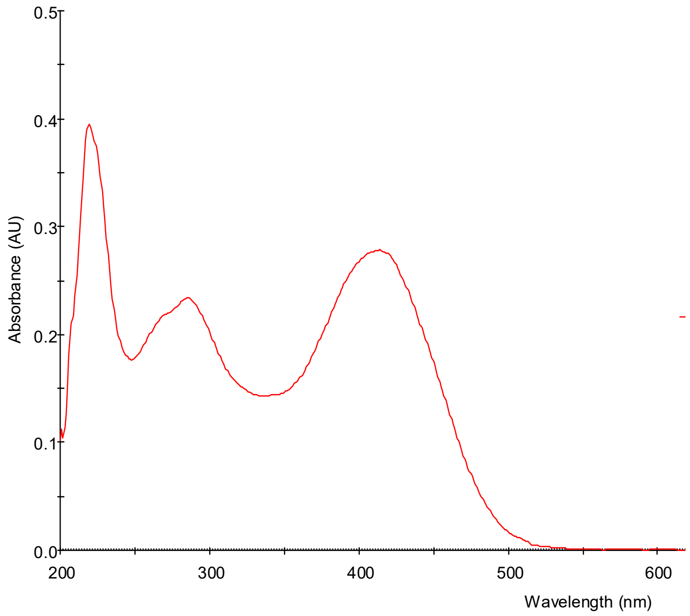

Figure S2: UV/VIS spectrum of **2**

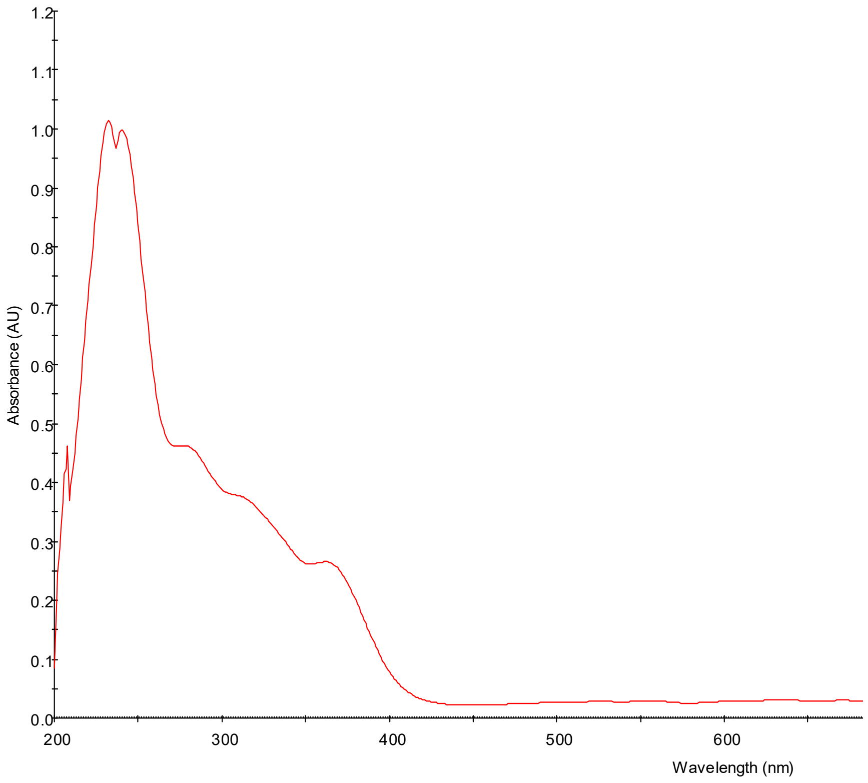

Figure S3: FTIR spectrum of **1**

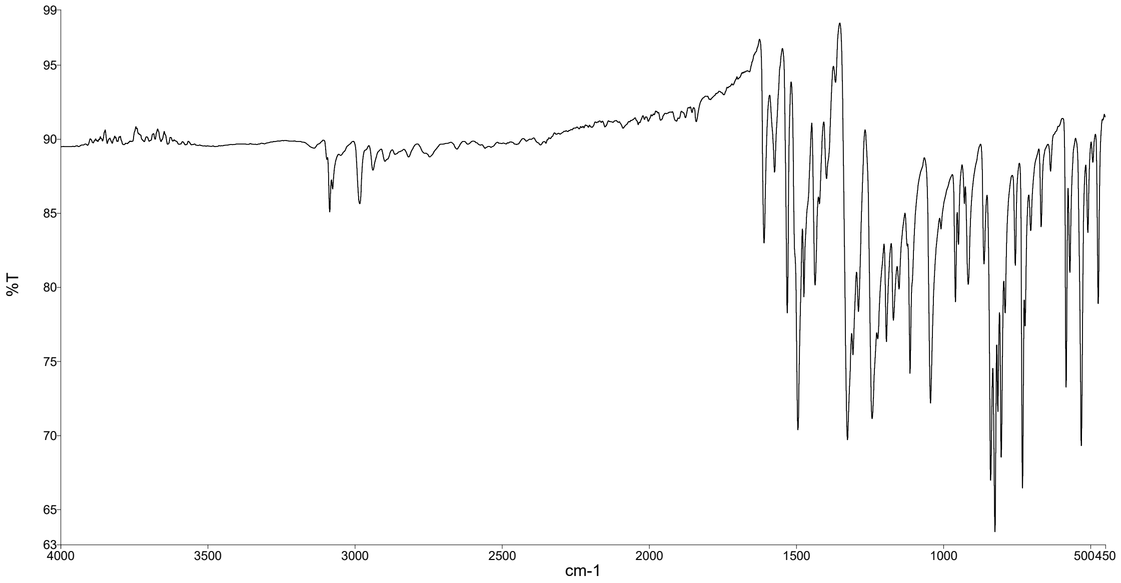

Figure S4: FTIR spectrum of **2**

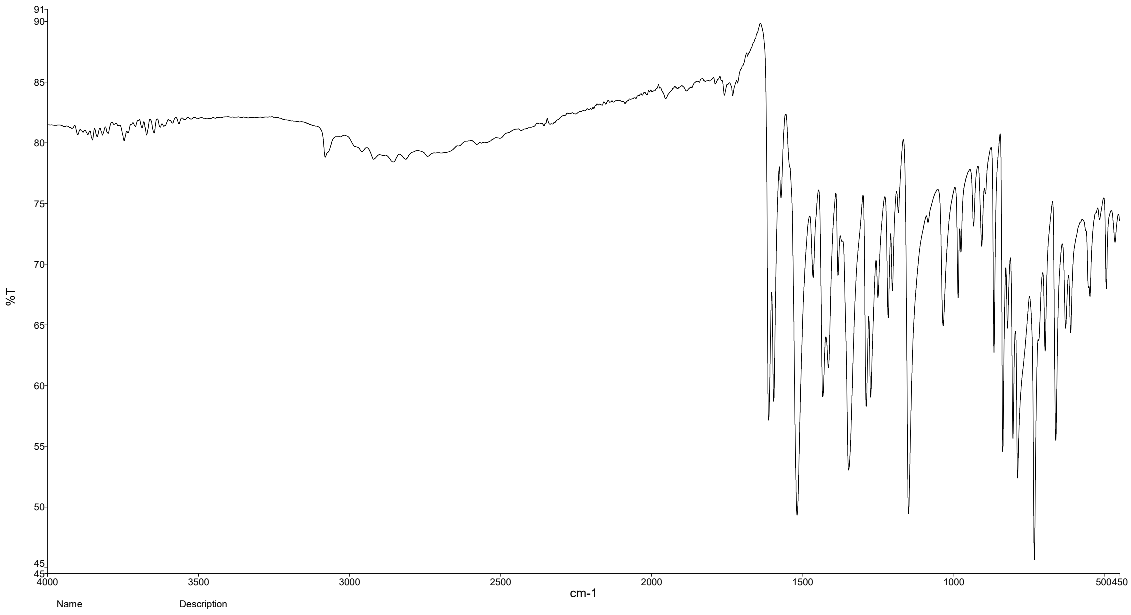

Figure S5: ^{1}H NMR spectrum of **1**

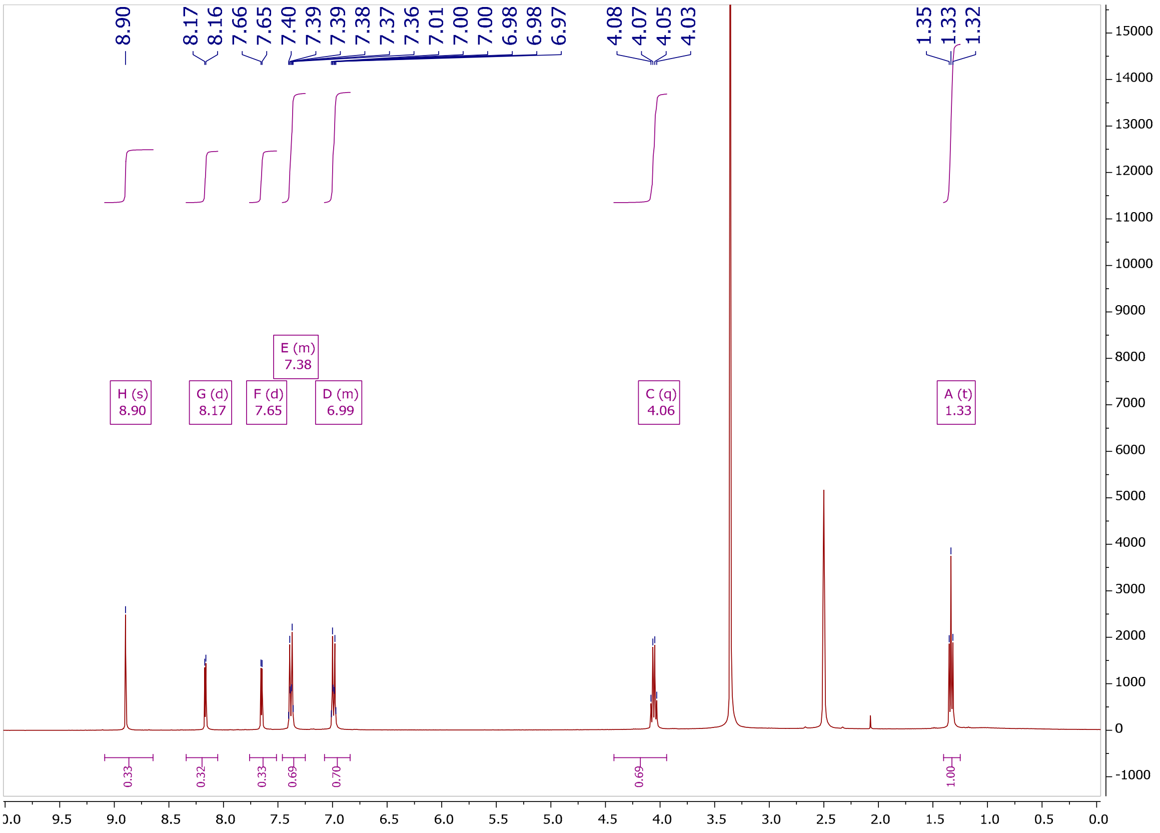

Figure S6: ^{1}H NMR spectrum of **2**

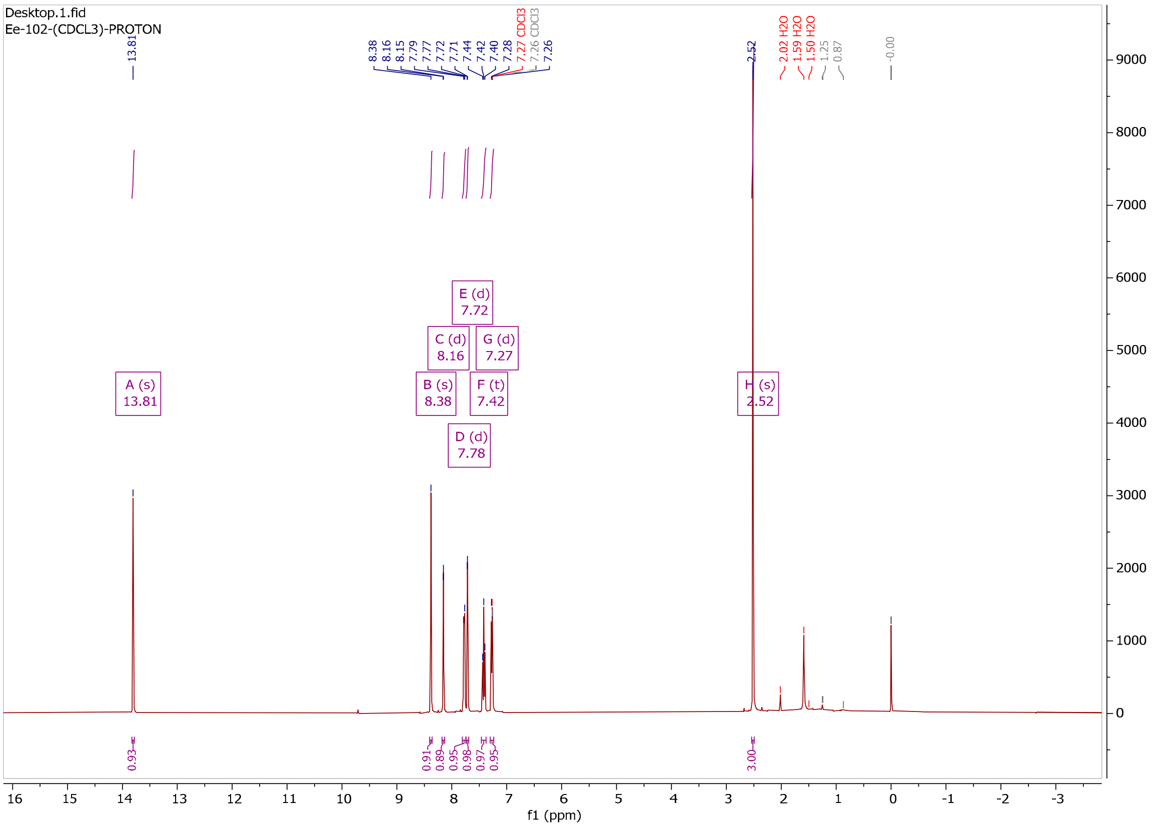

Figure S7: ^{13}C NMR spectrum of **1**

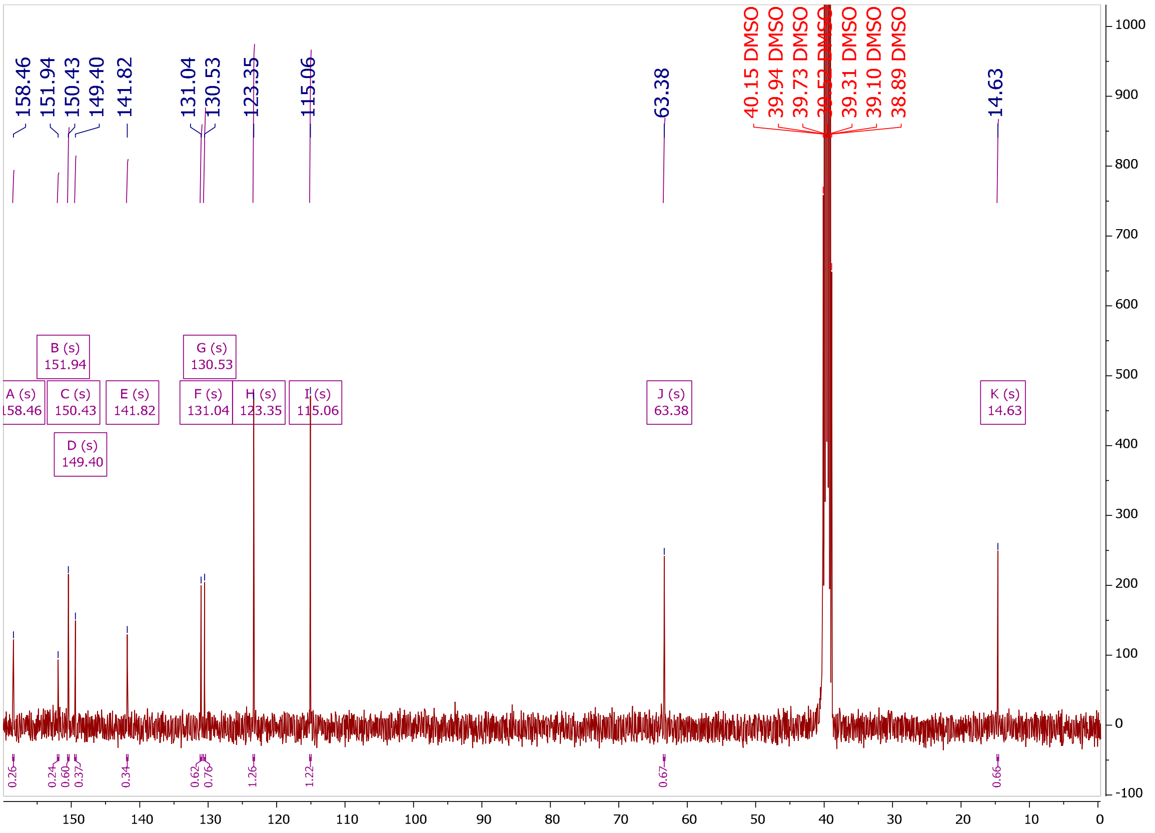

Figure S8: ^{13}C NMR spectrum of **2**

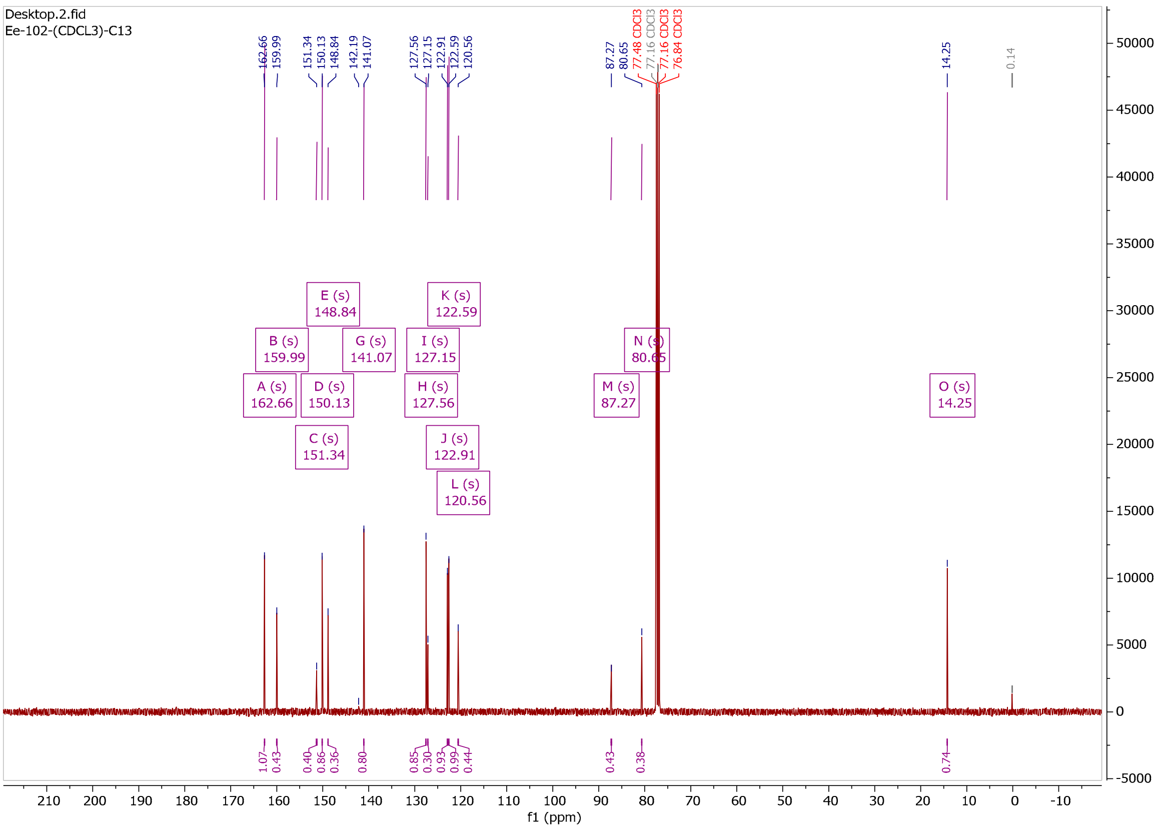

Table S1
Fractional Atomic Coordinates (×10^4) and Equivalent Isotropic Displacement Parameters (Å^2×10^3) for 1. U_{eq} is defined as 1/3 of of the trace of the orthogonalised U_{IJ} tensor.

Atom	*x*	*y*	*z*	U(eq)
S1	7903.9(7)	3758.3(6)	7595.1(9)	52.6(3)
O3	3095.7(19)	3840.3(16)	-2167(2)	53.2(6)
N2	6313(2)	3890.1(18)	4047(3)	47.8(6)
O2	10704(3)	3776(3)	11047(4)	97.2(11)
N1	9685(3)	3732(2)	10383(3)	67.2(8)
C9	3940(3)	3829(2)	-695(3)	43.9(6)
C6	5531(3)	3870(2)	2430(3)	45.6(7)
O1	8931(3)	3669(3)	11171(3)	101.4(12)
C10	3666(3)	4273(2)	708(4)	50.5(7)
C8	4998(3)	3393(2)	-530(3)	51.2(7)
C7	5787(3)	3410(2)	1022(4)	51.5(7)
C11	4453(3)	4283(2)	2253(3)	49.3(7)
C5	7373(3)	3892(2)	4077(4)	54.3(8)
C4	8262(3)	3845(2)	5652(4)	50.4(7)
C12	3381(3)	3405(3)	-3636(3)	54.8(8)
C1	9335(3)	3762(2)	8562(4)	53.7(8)
C13	2328(3)	3420(3)	-5068(4)	65.5(9)
C2	10061(3)	3817(3)	7481(4)	64.2(9)
C3	9415(3)	3862(3)	5788(4)	62.6(9)

Table S2

Anisotropic Displacement Parameters (Å^2×10^3) for 1. The Anisotropic displacement factor exponent takes the form: $-2\pi^2[h^2a^{*2}U_{11}+2hka^*b^*U_{12}+\ldots]$.

Atom	U_{11}	U_{22}	U_{33}	U_{12}	U_{13}	U_{23}
S1	44.6(5)	68.5(5)	45.2(4)	1.8(4)	11.6(3)	-5.0(3)
O3	49.2(13)	66.0(14)	41.8(10)	8.9(10)	5.3(9)	-4.0(9)
N2	47.6(16)	52.7(15)	40.9(12)	-0.2(11)	5.9(10)	3.7(9)
O2	70(2)	154(3)	56.3(15)	3.3(19)	-8.5(13)	-10.4(15)
N1	64(2)	92(2)	43.5(14)	3.2(16)	7.5(13)	-9.1(13)
C9	46.1(16)	44.4(15)	39.9(13)	3.1(12)	6.9(11)	2.0(10)
C6	46.2(17)	49.0(16)	40.6(13)	0.9(12)	8.1(11)	5.2(11)
O1	85(2)	174(4)	49.9(14)	-1(2)	24.6(14)	-7.2(16)
C10	42.8(17)	58.8(18)	49.9(15)	6.7(14)	10.5(12)	-6.1(13)
C8	52.3(18)	59.4(17)	41.1(13)	11.4(14)	9.2(12)	-5.4(12)
C7	47.5(17)	55.7(17)	50.0(15)	12.3(14)	8.1(12)	-1.7(12)
C11	46.4(17)	60.0(18)	41.9(14)	3.0(13)	10.7(12)	-3.9(12)
C5	53(2)	65(2)	45.1(15)	-1.0(14)	10.9(13)	-0.0(12)
C4	48.9(18)	59.5(18)	41.7(14)	3.2(13)	7.8(12)	-1.6(12)
C12	57(2)	66(2)	40.9(14)	5.9(15)	10.6(12)	-3.8(13)
C1	48.7(18)	69.1(19)	41.6(14)	1.1(15)	7.0(12)	-5.9(13)
C13	60(2)	81(2)	50.5(17)	6.5(18)	2.5(15)	-11.0(15)
C2	45.1(19)	94(3)	51.8(17)	2.0(17)	7.5(14)	-2.8(16)
C3	49.0(19)	90(3)	50.0(17)	-0.1(17)	13.9(13)	2.3(15)

Table S3

Torsion Angles for 1.

A	B	C	D	Angle/°	A	B	C	D	Angle/°
S1	C4	C5	N2	1.4(3)	N2	C6	C11	C10	-179.5(3)
S1	C4	C3	C2	-0.7(3)	N2	C5	C4	C3	-178.7(3)
S1	C1	N1	O2	-176.9(3)	O2	N1	C1	C2	1.4(5)
S1	C1	N1	O1	2.7(4)	N1	C1	C2	C3	-178.2(4)
S1	C1	C2	C3	0.2(3)	C9	C10	C11	C6	1.2(4)
O3	C9	C10	C11	178.8(3)	C9	C8	C7	C6	-0.5(4)
O3	C9	C8	C7	-179.0(3)	C5	C4	C3	C2	179.3(4)
N2	C6	C7	C8	179.1(3)	C4	C3	C2	C1	0.3(4)

Table S4

Hydrogen Atom Coordinates (Å×10[4]) and Isotropic Displacement Parameters (Å[2]×10[3]) for 1.

Atom	*x*	*y*	*z*	U(eq)
H10	2950(3)	4563(2)	604(4)	60.6(9)
H8	5182(3)	3088(2)	-1459(3)	61.4(9)
H7	6498(3)	3109(2)	1129(4)	61.9(9)
H11	4257(3)	4571(2)	3189(3)	59.2(9)
H5	7600(3)	3924(2)	3050(4)	65.2(9)
H12a	4000(3)	3762(3)	-3955(3)	65.8(9)
H12b	3636(3)	2746(3)	-3383(3)	65.8(9)
H13a	2517(7)	3170(20)	-6081(10)	98.2(14)
H13b	1739(10)	3026(17)	-4775(16)	98.2(14)
H13c	2053(15)	4071(4)	-5260(30)	98.2(14)
H2	10861(3)	3825(3)	7811(4)	77.0(11)
H3	9748(3)	3900(3)	4854(4)	75.1(11)

Table S5

Fractional Atomic Coordinates (×10[4]) and Equivalent Isotropic Displacement Parameters (Å[2]×10[3]) for 2. U_{eq} is defined as 1/3 of the trace of the orthogonalised U_{IJ} tensor.

Atom	*x*	*y*	*z*	U(eq)
I2	9672.2(4)	6393.7(3)	2935.6(3)	62.67(13)
I1	6466.6(5)	9050.3(3)	5505.1(3)	64.69(14)
O1	5832(4)	6734(3)	6290(3)	62.5(10)
N1	5963(4)	4669(3)	6481(3)	48.9(9)
N2	3662(5)	2914(4)	9109(4)	63.7(12)
O3	3839(6)	3645(4)	9749(4)	90.8(15)
O2	2896(5)	2158(4)	9183(4)	89.6(15)
C1	6675(5)	6641(4)	5586(4)	46.3(10)
C6	7146(5)	5637(4)	5310(4)	43.8(9)
C2	7126(5)	7553(4)	5115(4)	46.9(10)
C7	6731(5)	4671(4)	5775(4)	48.2(11)
C3	7979(5)	7472(4)	4376(4)	47.9(11)
C5	8021(5)	5570(4)	4570(4)	47.2(10)
C8	5586(5)	3724(4)	6965(4)	46.0(10)
C13	4767(5)	3836(4)	7754(4)	45.0(10)
C12	4451(5)	2901(4)	8234(4)	48.3(11)
C4	8433(5)	6478(4)	4098(4)	46.6(10)
C14	4275(6)	4921(4)	8004(5)	59.4(13)
C11	4871(6)	1904(4)	7965(4)	56.5(13)
C9	6038(6)	2710(4)	6717(5)	58.1(13)
C10	5678(6)	1820(4)	7214(5)	60.6(14)

Table S6
Anisotropic Displacement Parameters (Å^2×10^3) for 2. The Anisotropic displacement factor exponent takes the form: $-2\pi^2[h^2a^{*2}U_{11}+2hka^*b^*U_{12}+\ldots]$.

Atom	U_{11}	U_{22}	U_{33}	U_{23}	U_{13}	U_{12}
I2	67.2(2)	65.1(2)	64.9(2)	9.46(17)	35.23(18)	5.70(17)
I1	84.5(3)	39.02(16)	74.9(3)	-1.87(15)	26.4(2)	11.05(16)
O1	74(2)	49.4(19)	77(3)	-0.2(18)	47(2)	7.2(18)
N1	59(2)	36.0(18)	57(2)	-1.0(16)	23(2)	-3.1(17)
N2	73(3)	62(3)	60(3)	18(2)	23(2)	2(2)
O3	128(4)	87(3)	69(3)	-4(3)	48(3)	-6(3)
O2	99(4)	76(3)	106(4)	26(3)	51(3)	-11(3)
C1	46(2)	43(2)	51(3)	-1.4(19)	16(2)	4.3(19)
C6	46(2)	40(2)	48(3)	1.5(18)	15.2(19)	1.4(18)
C2	49(3)	37(2)	55(3)	2.8(19)	13(2)	6.3(18)
C7	52(3)	43(2)	53(3)	-1.9(19)	18(2)	2.1(19)
C3	48(3)	42(2)	55(3)	7(2)	13(2)	0.1(19)
C5	57(3)	38(2)	51(3)	2.9(19)	21(2)	7(2)
C8	53(3)	37(2)	49(3)	1.6(19)	11(2)	-1.4(19)
C13	49(2)	41(2)	46(2)	-1.5(18)	11(2)	-3.3(18)
C12	56(3)	44(2)	44(2)	5.7(19)	8(2)	-1(2)
C4	52(3)	48(2)	45(2)	5.8(19)	20(2)	4(2)
C14	76(4)	43(2)	67(3)	-4(2)	33(3)	-2(2)
C11	73(3)	40(2)	57(3)	12(2)	13(3)	-5(2)
C9	76(4)	42(2)	62(3)	1(2)	29(3)	3(2)
C10	85(4)	36(2)	62(3)	3(2)	18(3)	2(2)

Table S7

Torsion Angles for 2.

A	B	C	D	Angle/°	A	B	C	D	Angle/°
I1	C2	C3	C4	-179.8(4)	C2	C1	C6	C7	179.4(5)
O1	C1	C6	C7	0.2(8)	C2	C1	C6	C5	-1.1(7)
O1	C1	C6	C5	179.8(5)	C2	C3	C4	I2	178.0(4)
O1	C1	C2	I1	-0.5(7)	C2	C3	C4	C5	-0.1(8)
O1	C1	C2	C3	-179.1(5)	C7	N1	C8	C13	178.3(5)
N1	C8	C13	C12	-178.1(4)	C7	N1	C8	C9	0.7(8)
N1	C8	C13	C14	3.0(7)	C7	C6	C5	C4	179.4(5)
N1	C8	C9	C10	178.7(5)	C5	C6	C7	N1	178.0(5)
N2	C12	C11	C10	-175.9(5)	C8	N1	C7	C6	-178.0(5)
O3	N2	C12	C13	-35.1(8)	C8	C13	C12	N2	176.9(5)
O3	N2	C12	C11	143.1(6)	C8	C13	C12	C11	-1.2(8)
O2	N2	C12	C13	147.9(5)	C8	C9	C10	C11	-0.1(10)
O2	N2	C12	C11	-33.9(7)	C13	C8	C9	C10	1.1(9)
C1	C6	C7	N1	-2.4(8)	C13	C12	C11	C10	2.2(9)
C1	C6	C5	C4	-0.2(8)	C12	C11	C10	C9	-1.5(9)
C1	C2	C3	C4	-1.2(8)	C14	C13	C12	N2	-4.4(8)
C6	C1	C2	I1	-179.6(4)	C14	C13	C12	C11	177.6(5)
C6	C1	C2	C3	1.8(8)	C9	C8	C13	C12	-0.5(7)
C6	C5	C4	I2	-177.3(4)	C9	C8	C13	C14	-179.3(5)
C6	C5	C4	C3	0.7(8)					

Table S8

Hydrogen Atom Coordinates (Å×10[4]) and Isotropic Displacement Parameters (Å[2]×10[3]) for 2.

Atom	*x*	*y*	*z*	U(eq)
H1	5663.52	5266.51	6669.09	59
H7	7032.23	4024.16	5554.21	58
H3	8251.29	8083.34	4064.49	57
H5	8329.2	4908.91	4392.6	57
H14A	5010.95	5287.45	8470.88	89
H14B	3497.74	4850.36	8347.85	89
H14C	4003.56	5317.13	7354.61	89
H11	4607.19	1299	8292.12	68
H9	6588.44	2644.56	6208.62	70
H10	5983.94	1156.37	7040.57	73

Table S9

Hydrogen Bonds for 2.

D	H	A	d(D-H)/Å	d(H-A)/Å	d(D-A)/Å	D-H-A/°
N1	H1	O1	0.86	1.93	2.612(5)	135.9
N1	H1	O1	0.86	1.93	2.612(5)	135.9
C7	H7	O2[1]	0.93	2.57	3.419(6)	152.1
C5	H5	O2[1]	0.93	2.64	3.469(7)	148.5

[1]1/2+X,1/2-Y,-1/2+Z

Figure S9: Crystal Packing of Compound **1**

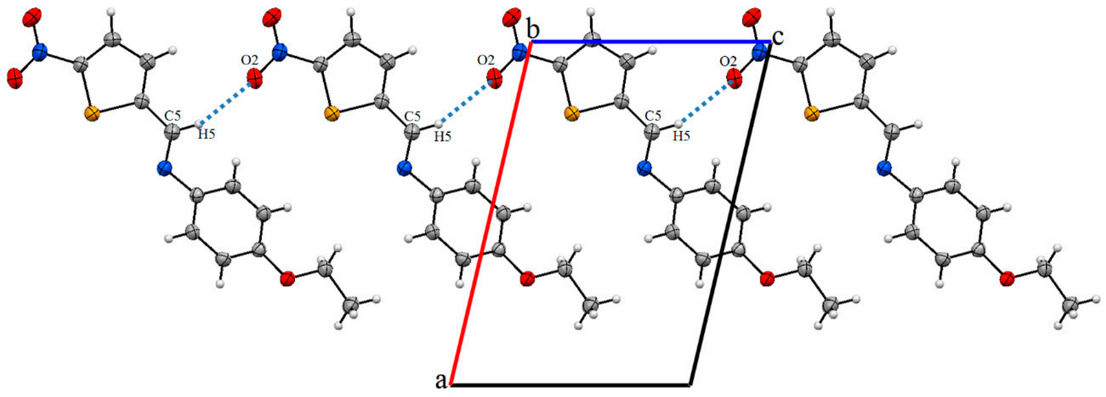

Figure S10: Crystal Packing of Compound **2**

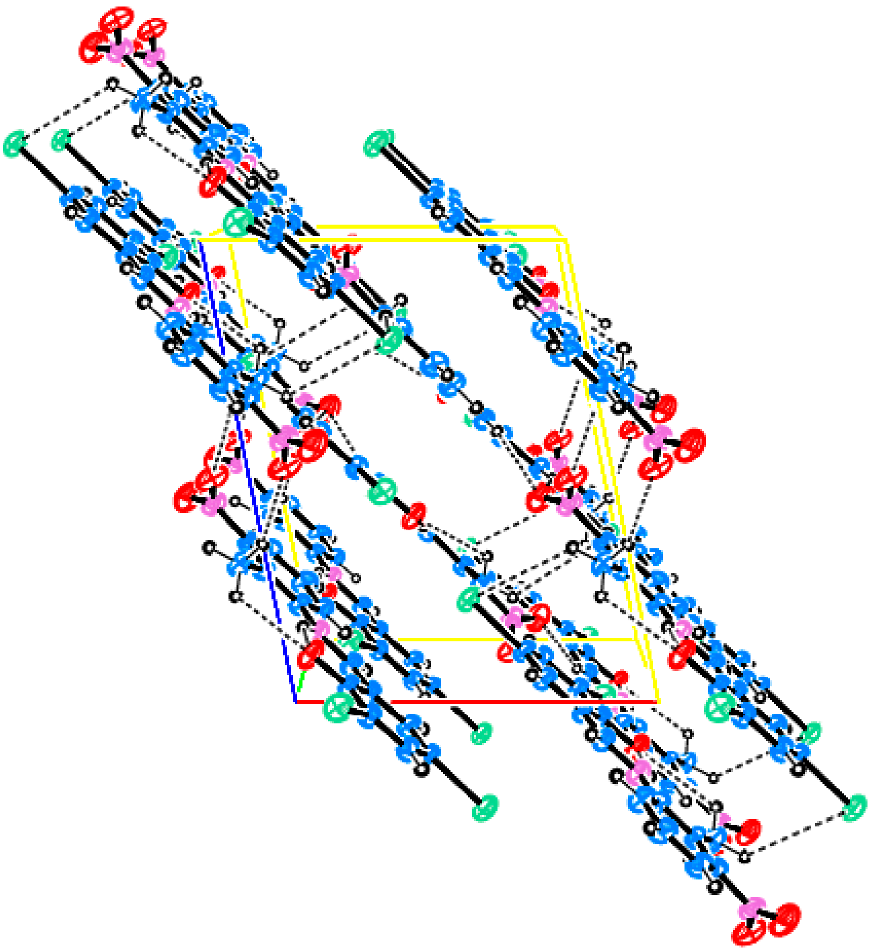

Table S10

Bond Lengths Compound 1

Atom	Atom	Length/Å		Atom	Atom	Length/Å	
		XRD	DFT			XRD	DFT
S1	C4	1.721(3)	1.73812	C9	C8	1.376(4)	1.39778
S1	C1	1.706(3)	1.73235	C6	C7	1.396(4)	1.39914
O3	C9	1.370(3)	1.35867	C6	C11	1.383(4)	1.40395
O3	C12	1.438(3)	1.43116	C10	C11	1.378(4)	1.37969
N2	C6	1.420(4)	1.39937	C8	C7	1.384(4)	1.38961
N2	C5	1.259(4)	1.27759	C5	C4	1.457(4)	1.44495
O2	N1	1.212(4)	1.22870	C4	C3	1.353(5)	1.38220
N1	O1	1.216(4)	1.23101	C12	C13	1.499(4)	1.51413
N1	C1	1.434(4)	1.43317	C1	C2	1.364(5)	1.36982
C9	C10	1.392(4)	1.40011	C2	C3	1.408(4)	1.40662

Table S11

Bond Angles of Compound 1

Atom	Atom	Atom	Angle/°		Atom	Atom	Atom	Angle/°	
			XRD	DFT				XRD	DFT
C1	S1	C4	89.24(14)	89.56136	C7	C8	C9	119.9(3)	119.89418
C12	O3	C9	116.4(2)	119.07134	C8	C7	C6	120.9(3)	121.15071
C5	N2	C6	117.6(3)	121.90917	C10	C11	C6	120.9(3)	119.39443
O1	N1	O2	123.9(3)	125.12738	C4	C5	N2	122.9(3)	121.54416
C1	N1	O2	118.6(3)	117.69641	C5	C4	S1	121.0(2)	121.32121
C1	N1	O1	117.5(3)	117.17621	C3	C4	S1	112.7(2)	112.10314
C10	C9	O3	116.0(3)	115.91614	C3	C4	C5	126.3(3)	126.57561
C8	C9	O3	124.4(3)	124.68193	C13	C12	O3	108.4(3)	107.73185
C8	C9	C10	119.6(3)	119.39443	N1	C1	S1	119.7(2)	120.12208
C7	C6	N2	122.5(3)	124.63403	C2	C1	S1	114.9(2)	114.02110
C11	C6	N2	119.1(3)	117.14736	C2	C1	N1	125.4(3)	125.85681
C11	C6	C7	118.3(3)	118.18184	C3	C2	C1	109.7(3)	111.12436
C11	C10	C9	120.2(3)	121.06833	C2	C3	C4	113.5(3)	113.18981

Table S12

Bond Lengths of Compound 2

Atom	Atom	Length/Å		Atom	Atom	Length/Å	
		XRD	DFT			XRD	DFT
I2	C4	2.105(5)	2.14869	C6	C5	1.404(6)	1.40695
I1	C2	2.087(5)	2.13744	C2	C3	1.387(7)	1.38527
O1	C1	1.345(6)	1.34383	C3	C4	1.398(7)	1.40487
N1	C7	1.288(6)	1.30218	C5	C4	1.391(7)	1.38084
N1	C8	1.424(6)	1.41775	C8	C13	1.418(7)	1.41913
N2	O3	1.220(7)	1.28507	C8	C9	1.408(7)	1.40151
N2	O2	1.230(6)	1.28297	C13	C12	1.390(7)	1.40460
N2	C12	1.484(7)	1.47362	C13	C14	1.505(7)	1.51441
C1	C6	1.415(6)	1.42757	C12	C11	1.387(7)	1.39602
C1	C2	1.409(7)	1.40793	C11	C10	1.367(8)	1.38597
C6	C7	1.447(6)	1.44395	C9	C10	1.369(7)	1.39217

Table S13

Bond Angles of Compound 2

Atom	Atom	Atom	Angle/°		Atom	Atom	Atom	Angle/°	
			XRD	DFT				XRD	DFT
C7	N1	C8	123.2(4)	123.06302	C13	C8	N1	117.4(4)	118.38028
O3	N2	O2	122.6(5)	124.25984	C9	C8	N1	122.5(4)	120.77215
O3	N2	C12	119.2(5)	118.71172	C9	C8	C13	120.0(4)	120.82942
O2	N2	C12	118.1(5)	117.02842	C8	C13	C14	119.6(4)	119.46093
O1	C1	C6	121.4(4)	121.22843	C12	C13	C8	116.0(4)	116.61137
O1	C1	C2	120.3(4)	121.01701	C12	C13	C14	124.3(4)	123.92213
C2	C1	C6	118.3(4)	117.75446	C13	C12	N2	121.4(5)	122.39183
C1	C6	C7	120.9(4)	119.21050	C11	C12	N2	115.1(4)	115.24695
C5	C6	C1	119.8(4)	120.53955	C11	C12	C13	123.5(5)	122.36113
C5	C6	C7	119.2(4)	120.24290	C3	C4	I2	119.0(3)	119.69337
C1	C2	I1	119.6(3)	118.38294	C5	C4	I2	121.7(4)	120.31940
C3	C2	I1	119.2(3)	120.53071	C5	C4	C3	119.3(4)	119.98723
C3	C2	C1	121.2(4)	121.08621	C10	C11	C12	119.2(5)	120.05136
N1	C7	C6	122.9(4)	121.09665	C10	C9	C8	121.0(5)	120.80809
C2	C3	C4	120.4(4)	120.51167	C11	C10	C9	120.2(5)	119.33372
C4	C5	C6	121.0(4)	120.12063					

Table S14

NBO of the synthesized compounds

Compounds	Donor (i)	Acceptor (j)	E(2)[Kcal/mol]	E(j)E(i) (a.u)	F(I.J)(a.u)
**1**	S1-C17	C28	0.51	1.38	0.024
	S1-C23	C17-C28	0.55	1.33	0.024
	O2-C6	C6-C9	0.58	1.48	0.026
	O2-C20	C24	0.59	1.45	0.026
	N3-C7	C-18	0.93	1.64	0.035
	N3-C18	C7-C15	0.74	0.91	0.024
	O4-N5	C23	0.63	1.08	0.034
	C6-C11	C9	0.88	1.84	0.036
	S1	C23	2.60	1.57	0.057
	N3	C7	2.60	1.35	0.054
**2**	I1-C19	C12	1.77	1.73	0.050
	I2-C9	C17	1.12	1.49	0.037
	N3-C10	C8	1.42	2.18	0.050
	N3-C16	C10	1.87	1.78	0.051
	N4-O5	C18	1.17	2.21	0.046
	N4-O5	O6	11.87	0.17	0.074
	N4-C18	O6	0.52	2.77	0.034
	C7-C8	C9	0.53	1.83	0.028
	C7-C9	C8	1.87	2.02	0.055
	C8-C14	C19	0.72	1.82	0.033

Table S15

Global Reactivity Parameters of the synthesized compounds.

Compounds	x	µ	η	IP	EA	1/2η	ω
**1**	6.344	-6.344	1.950	8.294	4.395	0.256	10.319
**2**	4.510	-4.510	1.658	6.11688	2.852	0.302	12.173

Figure S11: Correlation of Bond Length between DFT and SCXRD of Compound **1**

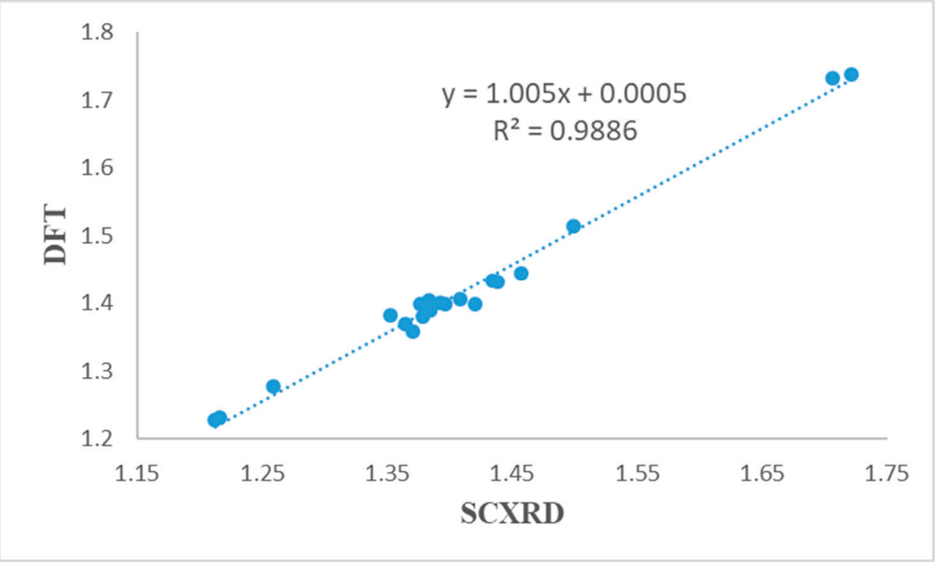

Figure S12: Correlation of Bond Angle between DFT and SCXRD of Compound **1**

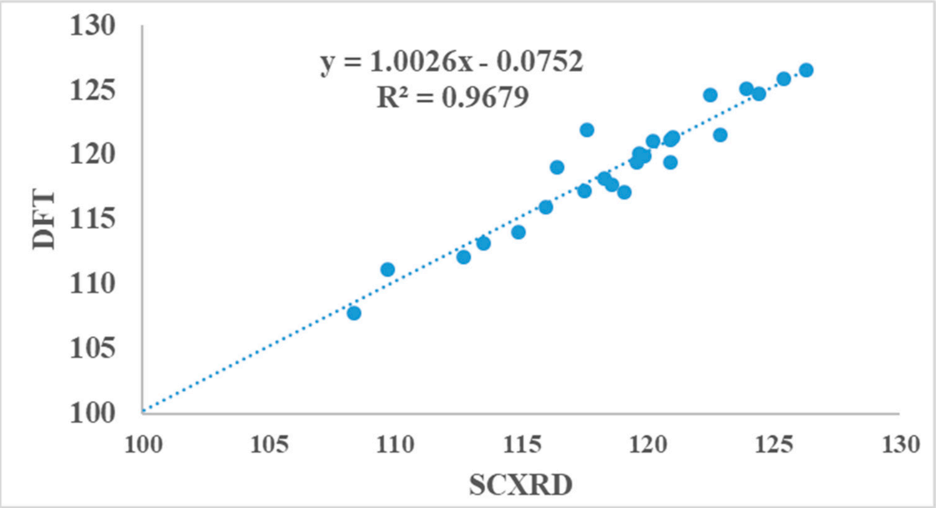

Figure S13: Correlation of Bond Length between DFT and SCXRD of Compound **2**

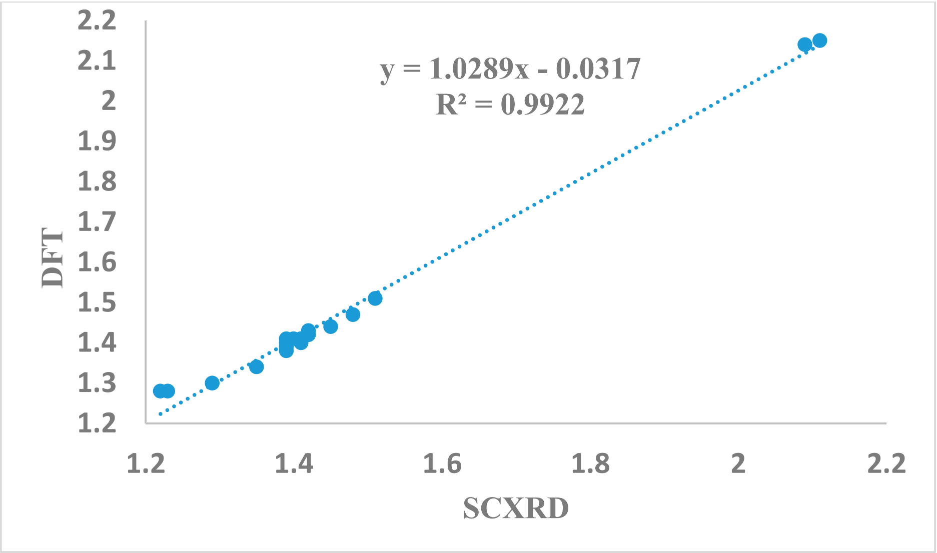

Figure S14: Correlation of Bond Angle between DFT and SCXRD of Compound **2.**
(**a**): Superimposed DFT and SCXRD structure of Compound **1;**
(**b**): Superimposed DFT and SCXRD structure of Compound **2.**

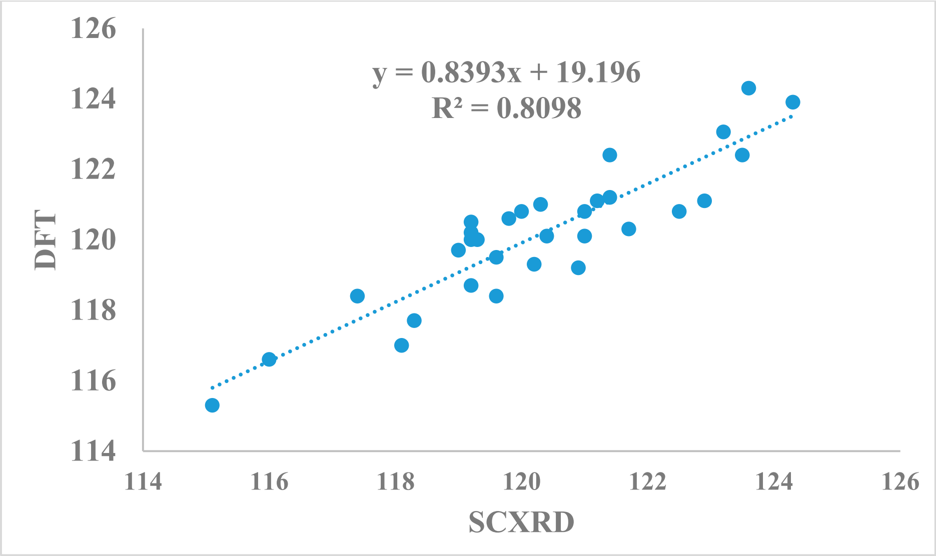

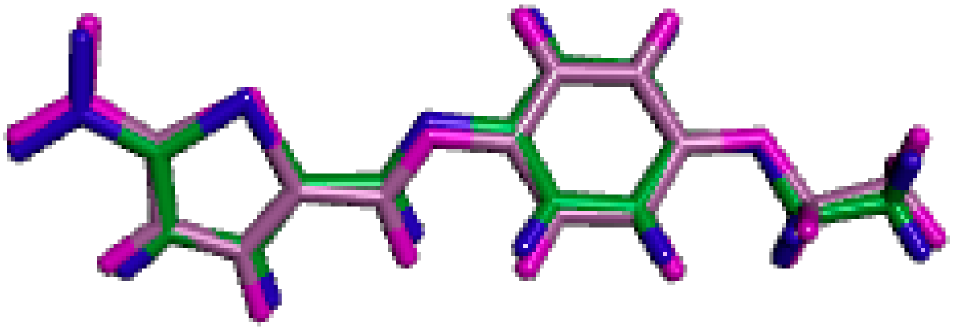

(**a**)

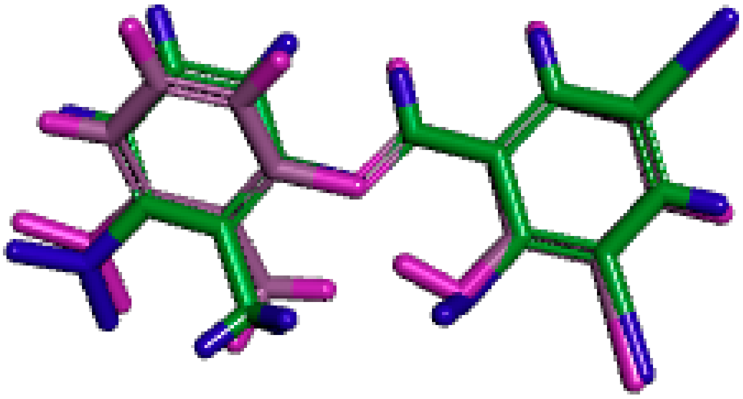

(**b**)

Figure S15: DOS spectra of the compounds **1** and **2**

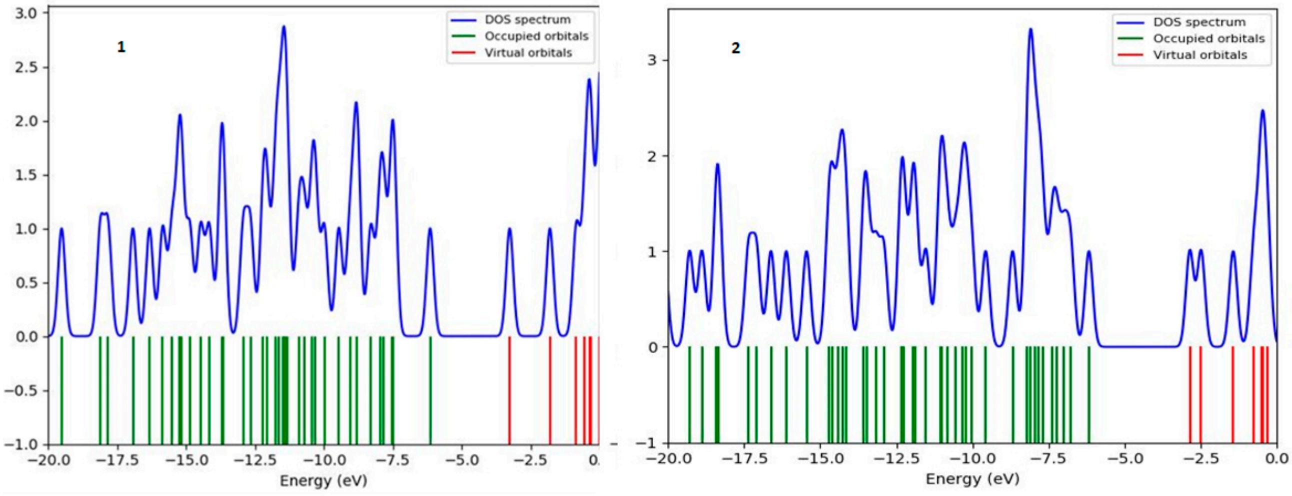

Figure S16: Fingerprint plots of contacts along with relative contributions for **1**

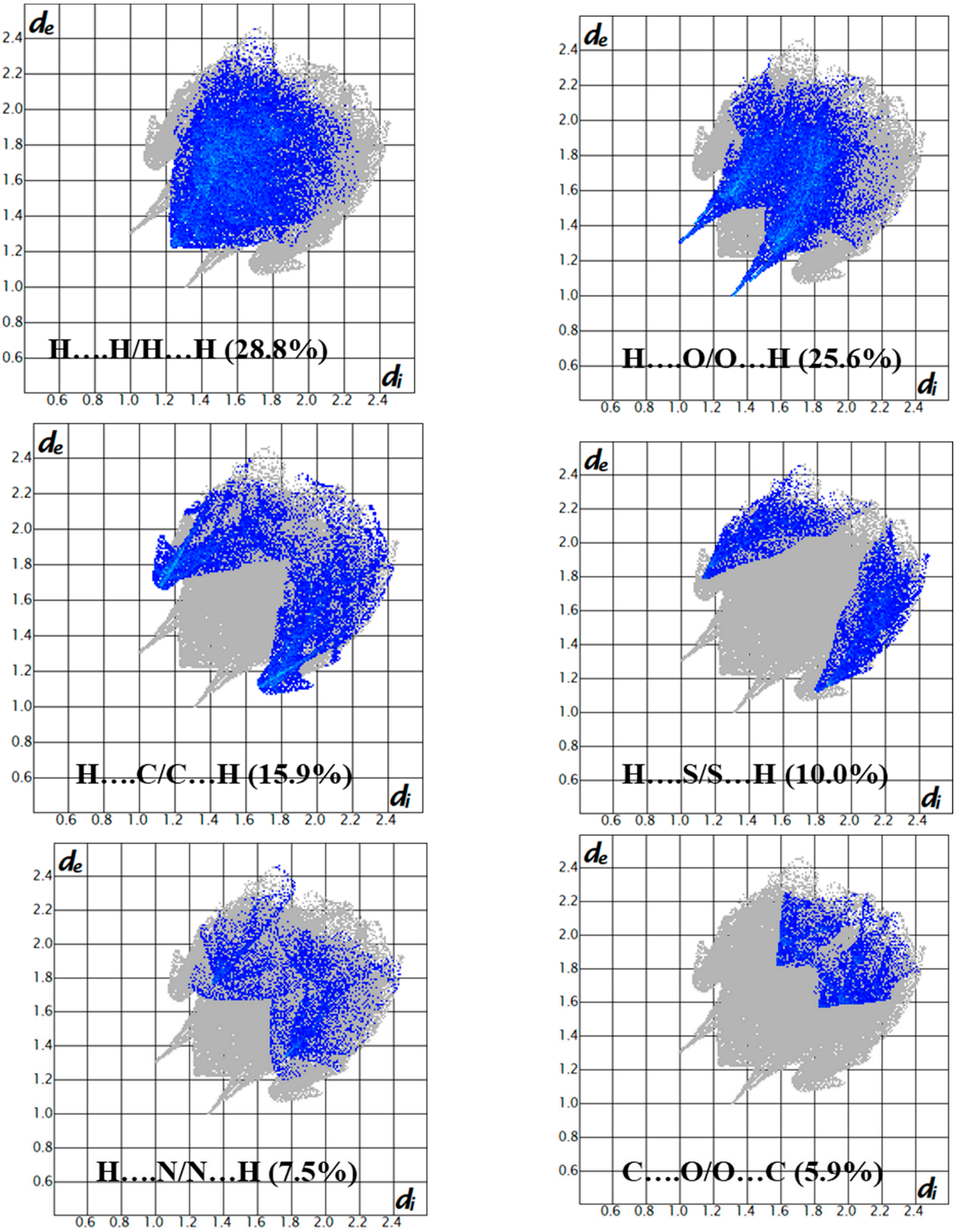

Figure S17: Fingerprint plots of contacts along with relative contributions for **2**

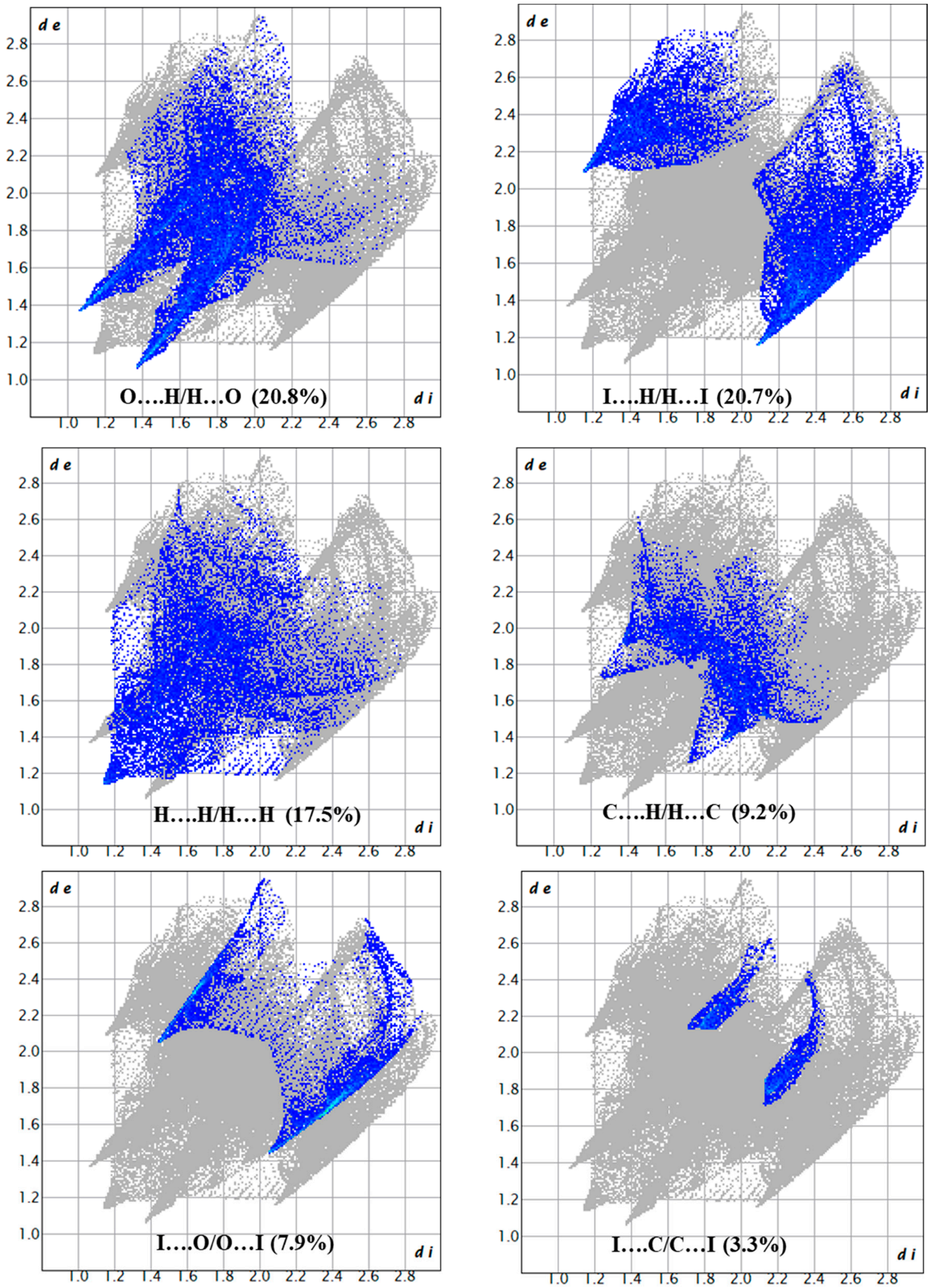

Figure S18: 3D Interactions of Compound **2** on Active Sites of AChE

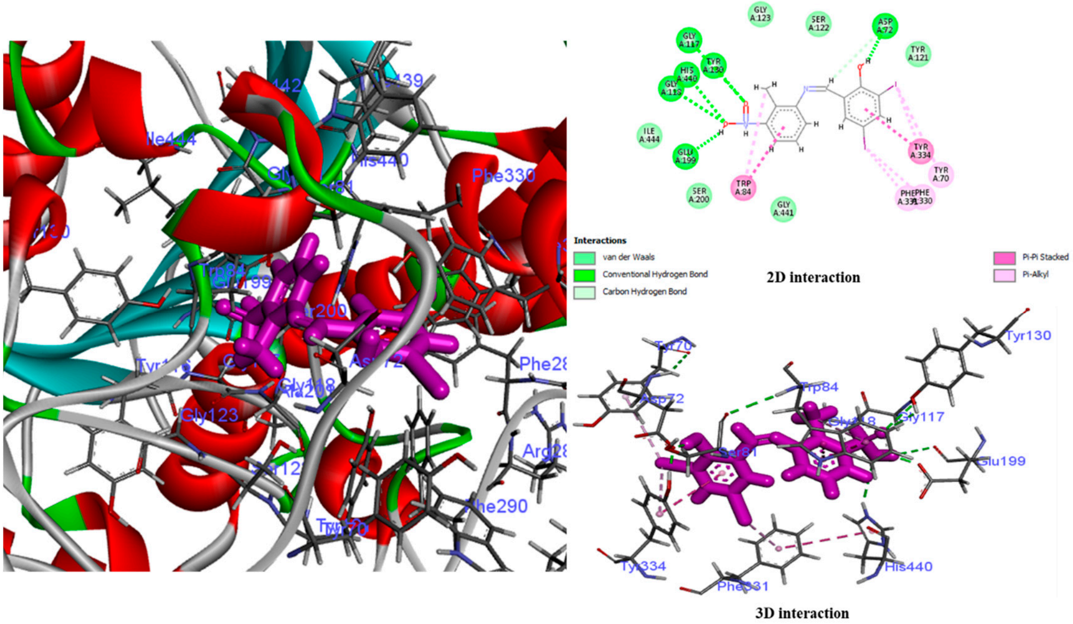

Figure S19: Docking Pose of Compound **1** with BChE

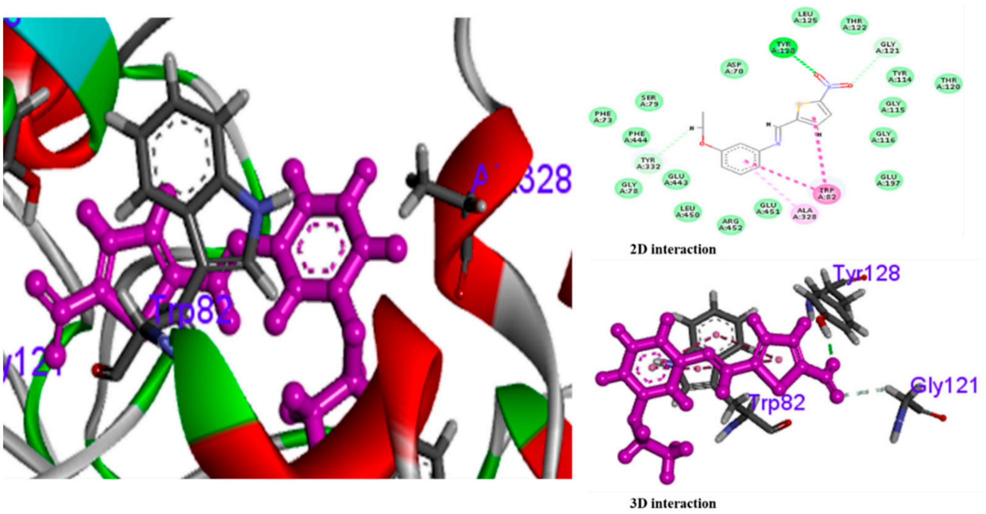

Figure S20: 3D Interactions of Compound **2** on Active Sites of BChE.

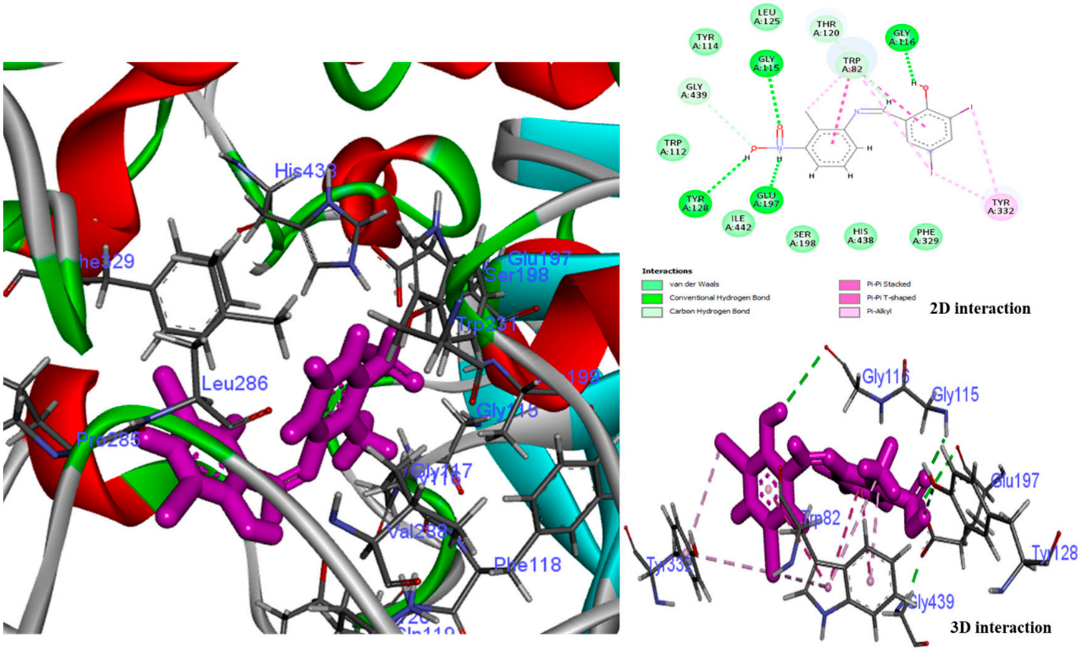

Supplement: Supplementary file 1 [file molecules-28-05703-s001.zip › molecules-2494342-supplementary.pdf]
